# Supplementary material for: Evaluating the impact on health outcomes of an event that resulted in a delay in contact tracing of COVID-19 cases in England, September 2020: an observational study
Source: BMJ Open. 2023 Oct 12;13(10):e064982. doi: 10.1136/bmjopen-2022-064982 (PMC10583033; doi:10.1136/bmjopen-2022-064982)
Supplement: Supplementary file 1 [file bmjopen-2022-064982.draft_revisions.pdf]

BMJ Open is committed to open peer review. As part of this commitment we make the peer review history of every article we publish publicly available.

When an article is published we post the peer reviewers' comments and the authors' responses online. We also post the versions of the paper that were used during peer review. These are the versions that the peer review comments apply to.

The versions of the paper that follow are the versions that were submitted during the peer review process. They are not the versions of record or the final published versions. They should not be cited or distributed as the published version of this manuscript.

BMJ Open is an open access journal and the full, final, typeset and author-corrected version of record of the manuscript is available on our site with no access controls, subscription charges or pay-per-view fees (http://bmjopen.bmj.com).

If you have any questions on BMJ Open's open peer review process please email info.bmjopen@bmj.com

BMJ Open

Evaluating the impact on health outcomes of an event that resulted in a delay in contact tracing of COVID-19 cases

Journal:	*BMJ Open*
Manuscript ID	bmjopen-2022-064982
Article Type:	Original research
Date Submitted by the Author:	23-May-2022
Complete List of Authors:	Findlater, Lucy; UK Health Security Agency South of England<br>Pierotti, Livia; University of Bristol<br>Turner, Charlie; UK Health Security Agency East of England<br>Wensley, Adrian; UK Health Security Agency North of England<br>Chen, Cong; UK Health Security Agency East of England<br>Seaman, Shaun; MRC Biostatistics Unit<br>Samartsidis, Pantelis; MRC Biostatistics Unit<br>Charlett, Andre; UK Health Security Agency<br>Anderson, Charlotte ; UK Health Security Agency<br>Hughes, Gareth; UK Health Security Agency North of England<br>Hickman, Matthew; University of Bristol<br>Edeghere, Obaghe; UK Health Security Agency Midlands and East of England<br>Oliver, Isabel; UK Health Security Agency South of England
Keywords:	COVID-19, EPIDEMIOLOGY, INFECTIOUS DISEASES, PUBLIC HEALTH

SCHOLARONE™
Manuscripts

I, the Submitting Author has the right to grant and does grant on behalf of all authors of the Work (as defined in the below author licence), an exclusive licence and/or a non-exclusive licence for contributions from authors who are: i) UK Crown employees; ii) where BMJ has agreed a CC-BY licence shall apply, and/or iii) in accordance with the terms applicable for US Federal Government officers or employees acting as part of their official duties; on a worldwide, perpetual, irrevocable, royalty-free basis to BMJ Publishing Group Ltd ("BMJ") its licensees and where the relevant Journal is co-owned by BMJ to the co-owners of the Journal, to publish the Work in this journal and any other BMJ products and to exploit all rights, as set out in our licence.

The Submitting Author accepts and understands that any supply made under these terms is made by BMJ to the Submitting Author unless you are acting as an employee on behalf of your employer or a postgraduate student of an affiliated institution which is paying any applicable article publishing charge ("APC") for Open Access articles. Where the Submitting Author wishes to make the Work available on an Open Access basis (and intends to pay the relevant APC), the terms of reuse of such Open Access shall be governed by a Creative Commons licence – details of these licences and which Creative Commons licence will apply to this Work are set out in our licence referred to above.

Other than as permitted in any relevant BMJ Author's Self Archiving Policies, I confirm this Work has not been accepted for publication elsewhere, is not being considered for publication elsewhere and does not duplicate material already published. I confirm all authors consent to publication of this Work and authorise the granting of this licence.

For peer review only

Title

Evaluating the impact on health outcomes of an event that resulted in a delay in contact tracing of COVID-19 cases

Authors

Lucy Findlater[1,2], Livia Pierotti[1,3], Charlie Turner[2], Adrian Wensley[2], Cong Chen[2], Shaun Seaman[4], Pantelis Samartsidis[4], Andre Charlett[1,2], Charlotte Anderson[2], Gareth Hughes[2], Matt Hickman[1,3], Obaghe Edeghere[2], Isabel Oliver[1,2,3]

Corresponding author: Lucy Findlater, UK Health Security Agency, 2 Rivergate, Bristol, UK, BS1 6EH. Email: lucy.findlater@phe.gov.uk. Phone: 01179069064

Affiliations

[1] National Institute of Health Research Health Protection Research Unit on Behavioural Science and Evaluation at the University of Bristol, Bristol, UK

[2] UK Health Security Agency, UK

[3] Population Health Sciences, Bristol Medical School, University of Bristol, UK

[4] MRC Biostatistics Unit, University of Cambridge, IPH Forvie Site, Robinson Way, Cambridge CB2 0SR, UK

Abstract

Objective*:* In September 2020, records of 15,861 SARS-CoV-2 cases failed to upload from the Second Generation Laboratory Surveillance System (SGSS) to the Contact Tracing Advisory Service (CTAS) tool, resulting in a delay in the contact tracing of these cases. This study used CTAS data to determine the impact of this delay on health outcomes: transmission events, hospitalisations, and mortality. Previously, a modelling study had suggested a substantial impact.

Design: Observational study

Setting: England.

Population: Individuals testing positive for SARS-CoV-2 and their reported contacts.

Main outcome measures: Secondary attack rates (SARs), hospitalisations, and deaths amongst primary and secondary contacts were calculated, compared to all other concurrent, unaffected cases. SGSS records affected by the event were matched to CTAS records and successive contacts and cases were identified.

Results: The initiation of contact tracing was delayed by 3 days on average in the primary cases in the delay group (6 days) compared to the control group (3 days). This was associated with lower completion of contact tracing of primary cases in the delay group: 80% (95%CI: 79-81%) in the delay group and 83% (95%CI: 83-84%) in the control group. There was some evidence to suggest an increase in transmission to non-household contacts amongst those affected by the delay. The SAR for non-household contacts was higher amongst secondary contacts in the delay group than the control group (delay group: 7.9%, 95%CI:6.4% to 9.2%; control group: 5.9%, 95%CI: 5.3% to 6.6%). There was no evidence of a difference between the delay and control groups in the odds of hospitalisation (crude odds ratio: 1.1 (95%CI: 0.9 to 1.2) or death (crude odds ratio: 0.7 (0.1 to 4.0)) amongst secondary contacts.

Conclusions: The delay in contact tracing had a limited impact on population health outcomes.

For peer review only - http://bmjopen.bmj.com/site/about/guidelines.xhtml

Strengths and limitations of the study

- Shows empirical data on the health impact of an event leading to a delay in contact tracing so can test hypotheses generated by models of the potential impact of a delay in contact tracing
- Estimates the extent of further transmission and odds of increased mortality or hospitalisation in up to the third generation of cases affected by the event
- The event acts as a natural experiment to describe the possible impact of contact tracing, comparing a group affected by chance by delayed contact tracing to a control group who experienced no delay
- Contact tracing was not completed for all individuals, so the study might not capture all affected contacts or transmissions

Introduction

As of April 2022, there have been over 21 million cases and 170,000 deaths from SARS-CoV-2 reported in the UK[1]. Contact tracing has been a central part of the public health response to SARS-CoV-2 and involves identifying contacts of people who have tested positive and advising them to self-isolate to reduce onward transmission of the virus[2]. On 28 May 2020, a national contact tracing system, NHS Test and Trace, was launched. All PCR positive cases in England were identified and and contacted by phone, digital tools, and through partnerships with local authorities[3 4]. Between 25 September and 2 October 2020, some data files containing test results from community testing sites failed to import from the laboratory surveillance system (Second Generation Surveillance System (SGSS)) to the contact tracing tool (Contact Tracing and Advisory Service (CTAS)). The files contained 15, 841 new positive cases[5]. At the time of the event, on the 28th September, there was a 7-day average of around 9500 reported cases per day in the UK with significant regional variation[1].

The event did not affect the notification of test results to patients and did not affect the results of any specific testing sites, geographical areas, or groups of the population. However, the delay in the upload of case records to CTAS resulted in a delay in the contact tracing of affected cases, and therefore a potential delay in instructing their contacts to self-isolate, which could have resulted in further transmission of SARS-CoV-2, and therefore potentially more COVID-19 related hospitalisations and deaths. We expect that health outcomes of the initial group of cases (primary cases) whose records failed to upload to CTAS would not have been affected by the event, as they received their test results and advice to isolate in the usual timeframe, and transmission events from primary cases to their contacts (primary contacts who may become secondary cases) would have most likely occurred prior to the isolation of the case. However, these primary contacts may not have been aware that they were a close contact of a case or advised to isolate in a timely manner, which may have increased the risk of transmission to their contacts (secondary contacts who may become tertiary cases). The outcomes of secondary contacts are therefore the most likely to have been impacted by the delay in contact tracing. In addition, there could potentially be delays in secondary cases seeking healthcare support, increasing their risk of hospitalisation or excess mortality.

A published preliminary model suggested that this event may have been associated with an increase of up to a third additional infections and 30 - 40% additional deaths[6].

For peer review only - http://bmjopen.bmj.com/site/about/guidelines.xhtml

In this study, we assess directly the health impact of the event, in terms of transmissions, hospitalisations, and deaths, using data on affected and concurrent cases and their contacts.

Aims and objectives

The aims of this study are to:

- Describe the nature of any contact tracing delay experienced by cases affected by the event.
- Assess the extent of further transmission, by calculating attack rates from primary cases to their contacts and from secondary cases to their contacts.
- Assess the impact of the delay on health outcomes of primary and secondary contacts, in terms of hospitalisations and deaths.

For peer review only

For peer review only - http://bmjopen.bmj.com/site/about/guidelines.xhtml

Methods

Data sources

The list of SGSS records known to have been affected by the event was provided by the UK Health Security Agency (UKHSA). SGSS contains demographic and diagnostic information from laboratory test reports for patients who tested positive for SARS-CoV-2[7]. SGSS records were matched to COVID-19 testing and contact tracing records from the Contact Tracing Advisory Service (CTAS) database to validate the cases affected by the event. CTAS records represent SARS-CoV-2 case episodes, including information on the movements of cases in their infectious period, their contacts, and demographic and clinical characteristics (Supplementary Figure 1, Supplementary Material)[8]. Matching was conducted in repeated rounds based on combinations of the following identifiers: SGSS unique identifier; NHS number; forename; surname; date of birth; and postcode. This data set of CTAS records constituted the primary cases affected by the event, described henceforth as the 'delay group'. A control group consisting of all primary cases from the same time window (30th September to 5th October 2020 inclusive) that were unaffected by the event was used as a comparison. The event affected exclusively cases arising from community testing sites and not hospital or other testing sites; therefore, primary cases in the control group were restricted to those arising from community testing[9].

Identifying cases and contacts

Contact records were linked to the case who reported them and assigned to the same group (delay or control group) as their associated case. Secondary cases were defined as (a) individuals reported as a contact by a primary case and (b) having a contact event with the primary case between 2 and 14 days inclusive prior to the onset of symptoms of the secondary case (or test date if no symptom onset available). If the secondary case was a household contact of the primary case, the date of interaction was taken to be the date of onset of symptoms (or test date if no symptom onset available) of the primary case. Secondary cases may be exposed to more than one primary case; therefore, only one transmission event was chosen to link a secondary case to the primary case who most likely infected them based on the following hierarchy: (1) a household transmission event was prioritised above all other types of contact event (due to the higher risk of transmission in this setting); (2) where multiple events of the same priority occurred, the most recent exposure was selected as the transmission event. Individuals who had taken multiple SARS-CoV-2 tests generated a CTAS record for each positive test result. These were identified by matching on name, DOB, NHS number, and contact details. For those cases, only the episode with the earliest test date was retained, as contact tracing should have been undertaken on receipt of the first positive result.

Linkage to health outcomes

Contact data sets were linked to mortality data provided by UKHSA which describe death in people with a positive SARS-CoV-2 test. Matching was performed using NHS number. Two definitions of mortality consistent with SARS-CoV-2 surveillance were used: 1) death of a person with a laboratory-confirmed positive SARS-CoV-2 test and whose death was within 60 days of the first specimen date or more than 60 days after the first specimen date if COVID-19 is mentioned on the death certificate, and 2) death of a person with a laboratory-confirmed positive SARS-CoV-2 test and whose death was within (equal to or less than) 28 days of the first positive specimen date[10].

Contact datasets were also linked to the UKHSA hospital onset COVID-19 data set (extracted on 22 November 2021), which pulls from the identifiable daily feeds of data on hospitalisations:

For peer review only - http://bmjopen.bmj.com/site/about/guidelines.xhtml

Secondary Uses Services (SUS+, a national data set describing patient hospitalisations); Emergency Care Data Set (ECDS, a national data set describing patient use of urgent and emergency care); and COVID-19 testing data. The data set provides one hospital spell per case if they had a hospital admission or discharge in the 14 day period before or after their first positive SARS-CoV-2 test, including people who were already in hospital at the time of test. Matching was performed using NHS number combined with date of birth, name, and sex.

Descriptive analysis

Analysis was performed in R version 1.3.1056. Demographic information and contact tracing outcomes were described for the case and contact data sets. Time taken for contact tracing was estimated using different dates in the contact tracing process: dates of test, laboratory report, SGSS record creation, CTAS record creation, and contact tracing completion (Supplementary Figure 2, Supplementary Material). Time taken to initiate the contact tracing process was defined as the days between the laboratory report date of the case and the date of upload of the case or contact record into CTAS. Time taken for the individual to complete contact tracing was defined as the days between the test date of the case and the date of contact tracing completion of the case or contact. Contact tracing completion was defined as the status of the individual's CTAS record being set to 'complete' by a call handler or the individual submitting an online contact tracing form.

Regression analysis

To understand the effect of the event on transmission, adjusted secondary attack rates (SARs) and differences in SARs amongst primary and secondary contacts were estimated via post-estimation from a logistic regression model. The predictor under investigation was whether the exposing case was in the delay or the control group. After review of crude SARs stratified by contact setting (household or non-household), effect modification of the predictor (delay or control group) by the contact setting (household or non-household) was allowed for in the model. The potential confounders included in the model were age group, sex, geographic region (one of nine UKHSA-defined geographical areas) of the contact, and whether the contact completed contact tracing.

To understand the effect of the event on health outcomes, odds ratios for experiencing mortality or hospitalisation amongst primary and secondary contacts were calculated using logistic regression with Firth penalisation, comparing individuals in the delay and control groups. When individuals had several contact episodes with multiple cases, the earliest episode per individual was retained. A very small number of mortality events was reported; therefore, unadjusted, crude odds ratios are presented to avoid overfitting the regression model, and the covariate balance between the delay and control groups suggests any major confounding is unlikely. As a sensitivity analysis, the health outcomes of the contacts in delay group were compared to a subgroup of contacts in the control group who were contact traced rapidly, defined as within three days of the date of test of the associated case.

Patient and public involvement

Patients or the public were not involved in the design, or conduct, or reporting, or dissemination plans of this research.

Results

For peer review only - http://bmjopen.bmj.com/site/about/guidelines.xhtml

Sample characteristics

Overall, 15,861 SGSS records were identified as having been affected by the event and 15,467 (98%) were matched to a CTAS record (Supplementary Table 1, Supplementary Material). Following data cleaning, 15,285 (96%) primary cases affected by the delay remained eligible for inclusion in the study (Supplementary Figure 3, Supplementary Material). A control group of 43,742 concurrent primary cases was created, consisting of all CTAS records from the time period of 30th September 2020 to 5th October 2020 inclusive which were not affected by the event. Secondary and tertiary cases and primary and secondary contacts were identified in the CTAS database and demographic information is described in the supplementary material (Supplementary Tables 2 and 3, Supplementary Material).

Nature of contact tracing delay

For primary cases in the delay group, it took on average two days longer for their records to be uploaded to CTAS, which initiated contact tracing, after the date the laboratory reported their positive test result (Table 1, Figure 1),. Primary cases also took on average three days longer to complete contact tracing in the delay group (6 days, interquartile range (IQR): 4-7) than the control group (3 days, (IQR: 2-5)) after their date of positive test. For primary contacts in the delay group, it took on average three days longer for their records to be uploaded to CTAS, relative to the laboratory report date of their associated case. It also took three days longer to complete contact tracing for primary contacts in the delay group (6 days (IQR: 5-8)) than the control group (3 days (IQR: 2-5)), relative to the test date of their associated case (Table 2, Figure 1). Once records had been uploaded onto CTAS, the primary cases and contacts in the delay group took the same median amount of time to complete contact tracing as the control group (1 day).

The proportion of cases for whom contact tracing was completed was slightly lower for primary cases in the delay group (80%, 95%CI: 79-81%) than the control group (83%, 95%CI: 83-84%), a difference of -3.5% (95%CI: -4.2% to -2.8%) (Table 1). There was no difference in the proportion of secondary and tertiary cases who completed contact tracing. The proportion of primary contacts traced was lower by -6.5% (95%CI: -7.1% to -5.9%) in the delay group than the control group (delay group 51%, 95%CI: 51-52%; control group 58%, 95%CI: 57-58%), but there was no evidence of a difference for secondary contacts (delay group 51%, 95%CI: 50-53%; control group 52%, 95%CI: 51-52%) (Table 2). For primary and secondary cases, the median numbers of household and non-household contacts per case was the same in the delay and control groups; for tertiary cases, the median number was the same for non-household contacts but was greater by one person for household contacts in the delay compared to the control group (Table 1). For contacts, the nature of contact was reported as household contact for 71-79% of contacts in each group (Table 2).

For peer review only - http://bmjopen.bmj.com/site/about/guidelines.xhtml

Table 1: Contact tracing outcomes for primary, secondary, and tertiary cases

	Delay group			Control group		
**Cases**	**Primary**	**Secondary**	**Tertiary**	**Primary**	**Secondary**	**Tertiary**
Number of observations	15,285	2748	382	43,742	9575	1335
Number of individuals	15,285 (100.0%)	2695 (98.1%)	375 (98.2%)	43,742 (100.0%)	9382 (98.0%)	1307 (97.9%)
Completed contact tracing (n (%, 95%CI))						
Yes	12,221 (80.0%, 79.3%-81%)	2137 (77.8%, 76.2%-79.3%)	276 (72.3%, 67.5%-76.7%)	36,503 (83.5%, 83.1%-83.8%)	7291 (76.1%, 75.3%-77.0%)	971 (72.7%, 70.3%-75.1%)
Difference in proportions who completed contact tracing (delay group minus control group) (95% CI)	-3.5% (-4.2% to -2.8%)	1.6% (-0.2% to 3.4%)	-0.5% (-5.7% to 4.8%)	N/A	N/A	N/A
Median (and IQR) number of contacts reported per case						
All	3 (1-4)	3 (1-4)	3 (1-5)	3 (1-4)	3 (1-4)	3 (1-4)
Household	2 (1-3)	2 (1-3)	3 (1-4)	2 (1-3)	2 (1-4)	2 (1-3)
Non-household	0 (0-1)	0 (0-0)	0 (0-0)	0 (0-1)	0 (0-0)	0 (0-0)
Mean number of contacts reported per case						
All	3.0	3.0	3.6	3.2	3.1	3.2
Household	2.2	2.4	2.6	2.3	2.5	2.4
Non-household	0.8	0.6	1.0	0.9	0.6	0.7
Number of cases with ≥2 household contacts (n (%, 95%CI))	7361 (48.2%, 47.4%-49.0%)	1348 (49.1%, 47.2%-50.9%)	182 (47.6%, 42.5%-52.8%)	22,244 (50.9%, 50.4%-51.3%)	4717 (49.3%, 48.3%-50.3%)	631 (47.3%, 44.6%-50.0%)
Median (and IQR) time taken for contact tracing, days between:						
SGSS lab report date to CTAS upload date	3 (2-4)	1 (0-2)	1 (0-1)	1 (0-1)	1 (0-1)	1 (0-1)
Test date to CTAS completion date	6 (4-7)	4 (2-5)	4 (2-5)	3 (2-5)	3 (2-5)	3 (2-5)

For peer review only - http://bmjopen.bmj.com/site/about/guidelines.xhtml

Mean time taken for contact tracing, time between:						
SGSS lab report date to CTAS upload date	3.4	1.3	1.1	1.0	0.8	0.8
Test date to CTAS completion date	6.1	4.1	4.2	3.5	3.7	3.7

Table 2: Contact tracing outcomes for primary and secondary contacts

	**Delay group**		**Control group**	
**Contacts**	**Primary**	**Secondary**	**Primary**	**Secondary**
Number of observations	37,373	6540	121,205	23,119
Number of individuals	34,047 (91.1%)	6045 (92.4%)	109,907 (90.7%)	21,201 (91.7%)
Completed contact tracing (n (%, 95%CI))				
Yes	19,072 (51.0%, 50.5%-51.5%)	3361 (51.4%, 50.2%-52.6%)	69,702 (57.5%, 57.2%-57.8%)	11,915 (51.5%, 50.9%-52.2%)
Difference in proportions who completed contact tracing (delay group minus control group) (95% CI)	-6.5% (-7.1% to -5.9%)	-0.1% (-1.5% to 1.2%)	N/A	N/A
Nature of contact (n (%, 95%CI))				
Household	27,477 (73.5%, 73.1%-74.0%)	5152 (78.8%, 77.8%-79.8%)	85,538 (70.6%, 70.3%-70.8%)	18,221 (78.8%, 78.3%-79.3%)
Non-household	9,635 (25.8%, 25.3%-26.2%)	1371 (21.0%, 20.0%-22.0%)	34,699 (28.6%, 28.4%-28.9%)	4609 (19.9%, 19.4%-20.5%)
NA	261 (0.7%)	17 (0.3%)	968 (0.8%)	289 (1.3%)
Median (and IQR) time taken for contact tracing, days between:				
SGSS lab report date of associated case and contact's upload date	4 (3-6)	2 (1-4)	1 (1-3)	1 (1-3)
Test date of associated case and	6 (5-8)	4 (3-6)	3 (2-5)	4 (3-5)

For peer review only

contact's completion date				
Mean time taken for contact tracing, days between:				
SGSS lab report date of associated case and contact's upload date	4.5	2.4	2.0	2.0
Test date of associated case and contact's completion date	6.4	4.4	3.9	4.2

For peer review only

For peer review only - http://bmjopen.bmj.com/site/about/guidelines.xhtml

Effect on transmission

There was no evidence of a difference between the delay and control groups in the overall adjusted secondary attack rates (SARs) in primary or secondary contacts (Table 3). However, differences were observed in the SARs when stratified by household or non-household setting; SARs in non-household secondary contacts were higher for the delay group (7.9%, 95% CI 6.5% to 9.2%) than the control group (5.9%, 95% CI 5.3% to 6.6%), representing an increase of 1.9% (95% CI 0.4% to 3.4%). No difference was observed between the groups amongst household secondary contacts. Household SARs in primary contacts were lower for the delay group than for the control group. Conversely, for non-household contacts, the SARs in primary contacts were higher for the delay group (6.3%, 95% CI 5.8% to 6.8%) than for the control group (5.7%, 95% CI 5.5% to 5.9%), an increase of 0.6% (95% CI 0.1% to 1.1%).

Effect on health outcomes

COVID-19 death was a rare outcome in both groups with no evidence of a difference in 28 or 60 day mortality rates amongst all primary or secondary contacts in the delay group compared to the control group (Table 4). Using the 28 day definition of mortality, for primary contacts, there were 2.9 deaths (95%CI: 1.4 – 5.4) per 10,000 in the delay group compared to 3.7 deaths (95%CI: 2.7 – 5.1) per 10,000 in the control group. For secondary contacts, this was 0.0 (95%CI: 0.0 – 6.1) deaths per 10,000 in the delay group compared to 1.4 (95%CI: 0.3 – 4.1) deaths per 10,000 in the control group. There was no evidence for a difference observed in the odds of mortality for the delay group compared to the control group (Table 4, Figure 2).

The proportion of contacts admitted to hospital as inpatients within 14 days of having their first COVID-19 test was almost identical in the delay and control groups, for both primary contacts (delay 3.3% (95%CI: 3.1-3.5%), control 3.1% (95%CI: 3.0-3.2%)) and secondary contacts (delay 3.4% (95%CI: 3.0-3.9%), control 3.2% (95%CI: 3.0-3.5%)) (Table 4), There was no significant difference in the crude or adjusted odds of hospital admission between the delay and control groups (Table 4, Figure 2). Additionally, there was no significant difference observed in the crude or adjusted odds of hospitalisation or death when the delay group was compared to a subset of the control group who were contact traced most rapidly (Supplementary Table 4, Supplementary Material).

For peer review only

For peer review only - http://bmjopen.bmj.com/site/about/guidelines.xhtml

Table 3: Adjusted secondary attack rates amongst contacts of primary and secondary cases in the delay group compared to the control group, by setting of contact

Setting of contact event	Delay			Control			Difference in attack rates (95% CI)**
	Number of contacts	Contacts becoming cases	Adjusted* secondary attack rate (SAR) (95% CI)	Number of contacts	Contacts becoming cases	Adjusted* secondary attack rate (SAR) (95% CI)	
**Primary contacts**							
All	37,373	2,702	7.5% (7.3 to 7.8%)	121,205	9,419	7.7% (7.5 to 7.8%)	-0.1% (-0.4 to 0.2%)
Household	27,477	2,063	8.1% (7.8 to 8.4%)	85,538	7,195	8.6% (8.4 to 8.7%)	-0.5% (-0.8 to -0.1%)
Non-household	9,896	639	6.3% (5.8 to 6.8%)	35,667	2,224	5.7% (5.5 to 5.9%)	0.6% (0.1 to 1.1%)
**Secondary contacts**							
All	6,540	377	5.8% (5.2 to 6.3%)	23,119	1,315	5.7% (5.4 to 6.0%)	0.1% (-0.6 to 0.7%)
Household	5,152	256	5.1% (4.5 to 5.7%)	18,221	990	5.6% (5.3 to 6.0%)	-0.5% (-1.2 to 0.2%)
Non-household	1,388	121	7.9% (6.5% to 9.2%)	4,898	325	5.9% (5.3 to 6.6%)	1.9% (0.4 to 3.4%)

*Adjusted SARs allowing for effect modification of the study type by household, and adjusted for the age group, sex and geographic region of the contact, and whether they completed contact tracing

**Difference in attack rates calculated by subtracting control SAR from delay SAR

Table 4: Odds ratios for mortality and hospital outcomes in the delay group compared to the control group

	Delay group		Control group		
**Primary contacts**	**Number (n = 34,047)**	**Proportion**	**Number (n = 109,907)**	**Proportion**	**Crude odds ratio***
Deaths 28 day definition	10	2.9 (1.4 – 5.4) per 10,000 population	41	3.7 (2.7 – 5.1) per 10,000 population	0.8 (0.4 to 1.6)
Deaths 60 day definition	10	2.9 (1.4 – 5.4) per 10,000 population	61	5.6 (4.2 – 7.1) per 10,000 population	0.6 (0.3 to 1.1)
Admission to hospital as inpatient	1121	3.3% (3.1% – 3.5%)	3364	3.1% (3.0% - 3.2%)	1.1 (1.0 to 1.2)
**Secondary contacts**	**Number (n = 6045)**	**Proportion**	**Number (n = 21,201)**	**Proportion**	**Crude odds ratio***
Deaths 28 day	0	0.0 (0.0 – 6.1) per 10,000	3	1.4 (0.3 – 4.1) per 10,000	0.5 (0.0 to 9.7)

For peer review only - http://bmjopen.bmj.com/site/about/guidelines.xhtml

For peer review only

definition		population		population	
Deaths 60 day definition	1	1.7 (0.0 – 9.2) per 10,000 population	7	3.3 (1.3 – 6.8) per 10,000 population	0.7 (0.1 to 4.0)
Admission to hospital as inpatient	206	3.4% (3.0% – 3.9%)	687	3.2% (3.0% – 3.5%)	1.1 (0.9 to 1.2)

*The control group is the reference group (odds ratio = 1)

For peer review only

Discussion

Statement of principal findings

The contact tracing of 15,861 SARS-CoV-2 cases affected by the event was delayed by an average of three days. There was evidence that the delay was associated with a slight decrease in the proportion of cases and contacts who completed contact tracing. Despite the delay, the mean and median number of overall contacts and non-household contacts made per case was similar in the delay group and the control group, for primary, secondary, and tertiary cases, and the median number of household contacts per case was near-identical.

As expected, we observed a higher SAR from secondary to tertiary cases, for non-household contact events in the delay group. There was no evidence of a difference in the SARs for secondary to tertiary cases overall or for household contact events. We found no evidence of a difference in hospitalisation or mortality among primary or secondary contacts in the delay or control group using the standard definitions for COVID-19-related hospitalisation and mortality used for surveillance purposes in the UK.

Strengths and weaknesses

The event acted as a natural experiment whereby one group of individuals by chance experienced delayed contact tracing, enabling a comparison of their outcomes with concurrent, unaffected cases and contacts to assess the impact of contact tracing more generally. Having a a single national system collating all test results and a single national contact tracing system facilitated the study. Through record linkage we could identify successive generations of contacts and cases and describe key health outcomes associated with the delay in contact tracing; however, some of these outcomes were rare and it is possible that we did not have the power to detect a small difference in hospitalisation or mortality.

Secondary transmission could only be estimated using the contacts reported by cases who met the contact definition[11]. These would not include unknown contacts; however, the similarity in the number of reported contacts between the two groups suggests that unknown contacts are also likely to be similar. Contact tracing was not completed for a minority of cases in the delay and control groups, so there are likely to be further transmission events that are unknown and not described. People who do not engage in contact tracing differ from those who do in terms of ethnicity and socioeconomic status; however, this is unlikely to differ between the two groups[12 13 14]. Completion of contact tracing was slightly lower in the delay group. This could have potentially increased further the likelihood of worse outcomes in this group. It is important to note that there was an inherent lag in the initiation of contact tracing for all cases; therefore, the delay group was not compared to a perfect example of contact tracing, which may explain the lack of difference in observed health outcomes. To assess this, we conducted sensitivity analyses comparing the delay group to a subset of the control group who initiated contact tracing more quickly than the average for the control group, but we still did not detect a difference in health outcomes and the results were consistent with the main analysis. Some cases had multiple records, for example, if they had tested several times. For the analysis, we retained only the earliest CTAS episode. It is possible that some cases in the delay group reported subsequent positive tests soon after their first test which were not affected by the

For peer review only - http://bmjopen.bmj.com/site/about/guidelines.xhtml

event (for example, after being admitted to hospital). Therefore, these people might still have been contact traced in a timely manner. Finally, primary cases were defined as being either affected by the event (delay group) or from the same time period (control group). Because the event occurred over a period of a small number of days, during which time transmission events occurred, primary cases generated some secondary and tertiary cases which were also classed as primary cases themselves and may have been directly affected by the event. Therefore, transmission chains may have been affected at different stages.

Strengths and weaknesses in relation to other studies

A previous modelling exercise using aggregate published data projected substantial adverse health outcomes, describing a drastic rise in SARS-CoV-2 infections and deaths in areas most affected by the event and estimating over 125,000 additional infections and 1,500 additional COVID-19-related deaths[6]. Their rapid modelling approach described broader regional trends in infections and deaths using publicly available population surveillance data from the nearest calendar week to the event. This has the advantage of capturing all individuals possibly affected by the event, including any unknown or unreported contacts. However, it could lead to an overestimation of the health impact of the event. Our study described outcomes of the cases known to be affected by the event, which were a subset of the cases from 25 September – 2 October 2020, but the previous modelling study defined affected cases as all those with specimen dates from 20 – 27 September 2020; this might also lead to less accurate estimation of the health impact of the event. It is also important to note that the event occurred at a point in the pandemic when incidence of COVID-19 was increasing nationally and more rapidly in some regions[15 16].

Meaning of the study

Contacts of SARS-CoV-2 cases are at increased risk of infection, and this risk is higher among household contacts[17 18 19]. It is also known that significant numbers of infected people are asymptomatic and can transmit the virus[17 18 20]. Therefore, tracing all contacts of cases of COVID-19 to inform them of their exposure and give them advice on measures to reduce the risk of onwards transmission, has been a cornerstone of the pandemic response. This event offered an opportunity to assess the impact of contact tracing on health outcomes. Interestingly, few differences were observed between people impacted by the delay and other concurrent cases and contacts.

There was no difference in the number of contact events. This may reflect that people have generally maintained low levels of contacts throughout the pandemic[20]. We observed an increase in secondary transmission among non-household contacts but this was small. An increase in transmission in non-household contacts is to be expected if people are not aware of their exposure and therefore not isolating. We did not, however, observe any difference in SARs in household contacts, which may be explained by a more limited effect of self-isolation in preventing transmission within households. However, because many secondary cases were exposed to the virus in their household, their onward household attack rates could be lowered as their exposer would not become a case again within the time period studied.

For peer review only - http://bmjopen.bmj.com/site/about/guidelines.xhtml

Contact tracing is an effective tool to control transmission of infection[21 22 23]. Modelling studies have estimated the benefit from contact tracing and self-isolation to reduce SARS-CoV-2 transmission[24 25 26]. There is more limited evidence on the actual impact of the very large scale contact tracing undertaken during the pandemic. For contact tracing to be effective it needs to be timely and reach, as far as possible, all contacts[27 28 29]. The lack of a significant adverse health impact observed in our study could reflect the fact that there was an inherent delay in the tracing of COVID-19 cases. The lack of a difference may also reflect that, at the time, people generally were limiting their contact with others, with low numbers of contacts made by individuals in both groups[20]. Our study may also have lacked the power to detect a difference in the rare health outcome of mortality. Other factors that may have contributed include awareness of exposure status and need for self-isolation prior to official notification from the contact tracing system and a majority of contact events occurring in the household, where self-isolation is less likely to prevent transmission. Future research should evaluate the most effective approaches to conducting large scale contact tracing.

Acknowledgements

We acknowledge the UK Health Security Agency (UKHSA) for permitting and facilitating access to data. We acknowledge Anne-Marie O'Connell, Richard Dunn, Tom Clare, Fernando Capelastegui, Russell Hope, Simon Collin, and Efejiro Ashano (UKHSA) and James Thomas (NHS Digital) for assisting with data acquisition and linkage. We acknowledge Daniela De Angelis (MRC Biostatistics Unit, UKHSA) for contributing to project development and feedback.

Contributors

LF, LP, and IO contributed to conception and design of the work, analysis and interpretation of data, and drafted the manuscript. CT, AW, CC, CA, and GH contributed to design of the work, and acquisition, analysis, and interpretation of data. SS, PS, MH, and AC contributed to design of the work and interpretation of data. All authors revised the manuscript criticially and approved the final version of the manuscript.

Funding

The authors have not declared a specific grant for this research from any funding agency in the public, commercial or not-for-profit sectors. LF, LP, AC, MH, and IO acknowledge support from the NIHR Health Protection Research Unit in Behavioural Science and Evaluation at University of Bristol. PS was funded by the NIHR Programme Grants for Applied Research programme (grant RP-PG-0616-20008). SS was funded by UKRI (grant MC_UU_00002/10) and UKHSA. For the purpose of open access, the author has applied a Creative Commons Attribution (CC BY) licence to any Author Accepted Manuscript version arising.

Disclaimer

The views expressed are those of the author and not necessarily those of the NIHR, the Department of Health and Social Care, or the UKHSA.

Competing interests

No competing interests to declare.

For peer review only - http://bmjopen.bmj.com/site/about/guidelines.xhtml

Patient consent for publication

Not required

Ethics approval

Approval for the study was granted from the Public Health England (PHE) (now UKHSA) Research Ethics and Governance Group (REGG). R&D reference: R&D 431.

Data availability statement

The data that support this study were collected as part of a public health response and are considered sensitive and not made publicly available. Reasonable requests for access to anonymised data and data dictionary will be considered by the authors on request.

Figure legend and captions

Figure 1 title: Graphs describing the time taken to initiate and complete contact tracing of cases and contacts in the delay and control groups

Figure 1 legend: Graphs showing the time taken to initiate and complete contact tracing are displayed. The time taken to initiate contact tracing was defined as the number of days between the laboratory report date of the case and the CTAS upload date of the case (for cases) or the contact (for contacts). The time taken to complete contact tracing was defined as the number of days between the date of test of the case and the completion of contact tracing of the case (for cases) or the contact (for contacts).

Figure 2 title: Graph describing odds ratios for mortality and hospitalisation, comparing the delay group to the control group

Figure 2 legend: Crude odds ratios for mortality and hospitalisation are displayed. The control group is the reference group (odds ratio = 1). Death 28 days = death of a person with a laboratory-confirmed positive SARS-CoV-2 test and whose death was within 28 days of the first positive specimen date. Death 60 days = death of a person with a laboratory-confirmed positive SARS-CoV-2 test and whose death was within 60 days of the first specimen date or more than 60 days after the first specimen date if COVID-19 was mentioned on the death certificate. Admitted to hospital as inpatient = individual had a hospital admission or discharge in the 14 day period before or after their first positive SARS-CoV-2 test.

For peer review only - http://bmjopen.bmj.com/site/about/guidelines.xhtml

References

[1] UK Government (2021). Coronavirus (COVID-19) in the UK. UK summary. Available from https://coronavirus.data.gov.uk/. Accessed on 25.04.22

[2] Public Health England (2021) NHS Test and Trace Available from https://contact-tracing.phe.gov.uk/. Accessed on 01.12.21

[3] Public Health England (2020) Letter from Richard Gleave, Deputy Chief Executive of Public Health England, describing the use of CTAS in the COVID-19 response. Available from https://assets.publishing.service.gov.uk/government/uploads/system/uploads/attachment_data/file/881231/Letter_to_DsPH_on_contact_tracing.pdf. Accessed on 01.12.21

[4] Keeling et al. (2020) Efficacy of contact tracing for the containment of the 2019 novel coronavirus (COVID-19). J Epidemiol Community Health. 2020;74:861-866.

[5] Public Health England (2020). PHE statement on delayed reporting of COVID-19 cases. Available from https://www.gov.uk/government/news/phe-statement-on-delayed-reporting-of-covid-19-cases. Accessed on 01.12.21

[6] Fetzer and Graeber (2020). Does contact tracing work? Quasi-experimental evidence from an Excel error in England. CAGE working paper no. 521. Available from https://www.pnas.org/content/118/33/e2100814118. Accessed on 01.12.21

[7] NHS Digital (2021). SGSS and CHESS data. Available from: https://digital.nhs.uk/about-nhs-digital/corporate-information-and-documents/directions-and-data-provision-notices/data-provision-notices-dpns/sgss-and-chess-data. Accessed on 01.12.21

[8] UK Health Security Agency (2022). NHS Test and Trace in the workplace. Available from: https://www.gov.uk/guidance/nhs-test-and-trace-workplace-guidance. Accessed on 04.02.22

[9] Department of Health & Social Care (2020). COVID-19 testing data: methodology note. Available from https://www.gov.uk/government/publications/coronavirus-covid-19-testing-data-methodology/covid-19-testing-data-methodology-note. Accessed on 01.12.21

[10] Public Health England (2020). PHE data series on deaths in people with COVID-19: technical summary. Available from https://www.gov.uk/government/publications/phe-data-series-on-deaths-in-people-with-covid-19-technical-summary. Accessed on 01.12.21

[11] UK Health Security Agency (2022). (Withdrawn) Guidance for contacts of people with confirmed coronavirus (COVID-19) infection who do not live with the person. Available from: https://www.gov.uk/government/publications/guidance-for-contacts-of-people-with-possible-or-confirmed-coronavirus-covid-19-infection-who-do-not-live-with-the-person/guidance-for-contacts-of-people-with-possible-or-confirmed-coronavirus-covid-19-infection-who-do-not-live-with-the-person. Accessed on 10.05.22

[12] Shelby et al. (2021). Lessons learned from COVID-19 contact tracing during a public health emergency: a prospective implementation study. Frontiers in Public Health. 2021:9. doi: 10.3389/fpubh.2021.721952

[13] Munzert S, Selb P, Gohdes A, et al. Tracking and promoting the usage of a COVID-19 contact tracing app. Nat Hum Behav 2021;5(2):247-55. doi: 10.1038/s41562-020-01044-x

[14] Pegollo et al. (2021). Characteristics and determinants of population acceptance of COVID-19 digital contact tracing: a systematic review. Acta Biomedica. 2021;92(6):e2021444. doi: 10.23750/abm.v92iS6.12234

[15] Office for National Statistics (2020). Coronavirus (COVID-19) Infection Survey pilot: England, Wales and Northern Ireland, 2 October 2020. Available from: https://www.ons.gov.uk/peoplepopulationandcommunity/healthandsocialcare/conditionsanddiseases/bulletins/coronaviruscovid19infectionsurveypilot/englandwalesandnorthernireland2october2020. Accessed on 11.05.21.

[16] Office for National Statistics (2020). Coronavirus (COVID-19) Infection Survey pilot: England, Wales and Northern Ireland, 9 October 2020. Available from: https://www.ons.gov.uk/peoplepopulationandcommunity/healthandsocialcare/conditionsanddiseases/bulletins/coronaviruscovid19infectionsurveypilot/englandwalesandnorthernireland9october2020. Accessed on 11.05.21.

[17] Marchant et al. (2021). Determining the acceptability of testing contacts of confirmed COVID-19 cases to improve secondary case ascertainment. J Public Health (Oxf). 2021;43(3):446-452. doi: 10.1093/pubmed/fdab079

[18] Love et al. (2021). The acceptability of testing contacts of confirmed COVID-19 cases using serial, self-administered lateral flow devices as an alternative to self-isolation. medRxiv. doi: https://doi.org/10.1101/2021.03.23.21254168

[19] UK Health Security Agency (2021). Insights on transmission of COVID-19 with a focus on the hospitality, retail and leisure sector. Available from: https://assets.publishing.service.gov.uk/government/uploads/system/uploads/attachment_data/file/982865/S1194_Transmission_in_hospitality_retail_leisure.pdf. Accessed on 01.12.21.

For peer review only - http://bmjopen.bmj.com/site/about/guidelines.xhtml

[20] UK Health Security Agency (2021). Weekly statistics for NHS Test and Trace (England): 14 October to 20 October 2021. Available from: https://assets.publishing.service.gov.uk/government/uploads/system/uploads/attachment_data/file/1029536/test-and-trace-oct-14-to-20.pdf. Accessed on 01.12.21.
[21] Hossain et al. (2022). Effectiveness of contact tracing in the control of infectious diseases: a systematic review. The Lancet Public Health. 2022;7(3):e259-e273
[22] Bicher et al. (2021). Evaluation of Contact-Tracing Policies against the Spread of SARS-CoV-2 in Austria: An Agent-Based Simulation. Med Decis Making. 2021;41(8):1017-1032. doi: 10.1177/0272989X2110133062
[23] Fields et al. (2021). Coronavirus Disease Contact Tracing Outcomes and Cost, Salt Lake County, Utah, USA, March–May 2020. Emerging Infectious Diseases. 27(12):2999-3008. doi: https://doi.org/10.3201/eid2712.210505
[24] Kucharski et al (2020). Effectiveness of isolation, testing, contact tracing, and physical distancing on reducing transmission of SARS-CoV-2 in different settings: a mathematical modelling study. Lancet Infect Dis . 2020;20(10):1151-1160. doi: 10.1016/S1473-3099(20)30457-6.
[25] Chen and Huang (2022). Modelling the impacts of contact tracing on an epidemic with asymptomatic infection. Applied Mathematics and Computation. 2022;416. doi: https://doi.org/10.1016/j.amc.2021.126754
[26] Amaku et al (2021). Modelling the impact of contact tracing of symptomatic individuals on the COVID-19 epidemic. Clinics (Sao Paulo). 2021;76:e2639. doi: 10.6061/clinics/2021/e2639. eCollection 2021nh
[27] Ferretti et al. (2020). Quantifying SARS-CoV-2 transmission suggests epidemic control with digital contact tracing. Science. 2020;368(6491):eabb6936. doi: 10.1126/science.abb6936. Epub 2020 Mar 31.
[28] Grantz et al. (2021). Maximizing and evaluating the impact of test-trace-isolate programs: A modelling study. PLOS Medicine. 2021;18(4): e1003585. doi: https://doi.org/10.1371/journal.pmed.1003585
[29] Ashcroft P, Lehtinen S, Bonhoeffer S. Test-trace-isolate-quarantine (TTIQ) intervention strategies after symptomatic COVID-19 case identification. PloS one 2022;17(2):e0263597. doi: 10.1371/journal.pone.0263597

For peer review only

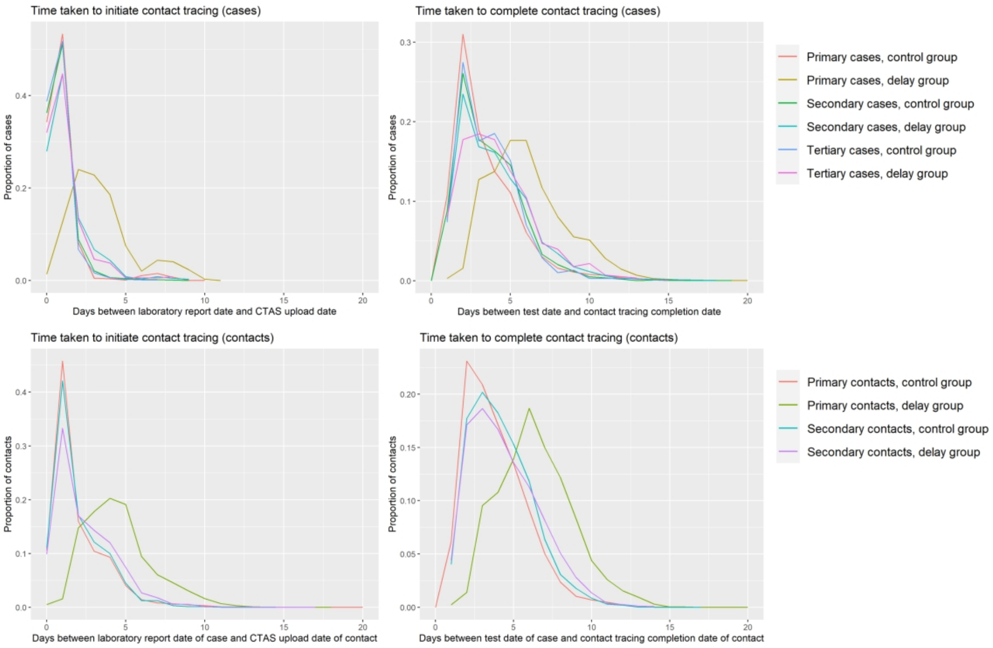

Figure 1: Graphs describing the time taken to initiate and complete contact tracing of cases and contacts in the delay and control groups

289x190mm (300 x 300 DPI)

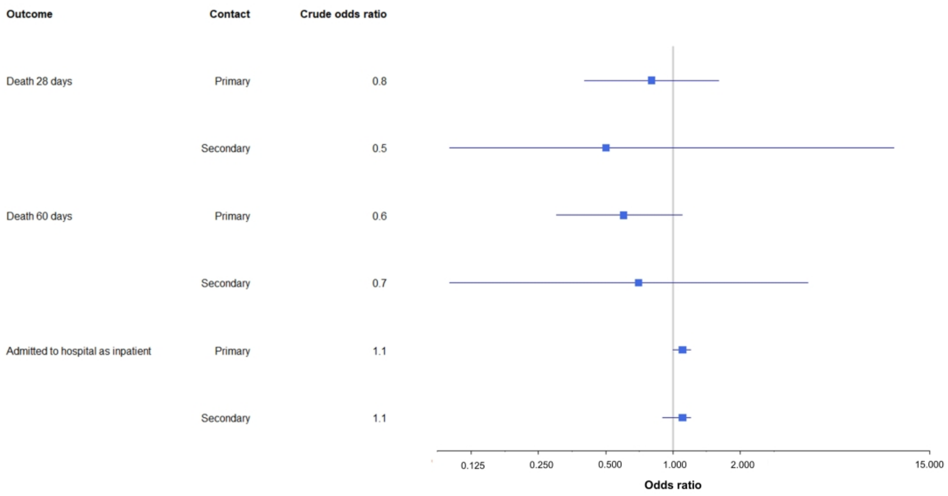

Figure 2: Graph describing odds ratios for mortality and hospitalisation, comparing the delay group to the control group (reference odds ratio = 1)

338x190mm (300 x 300 DPI)

Supplementary Material

Supplementary Figure 1: Definitions of SARS-CoV-2 cases and contacts

Definitions of cases and contacts in the Contact Tracing Advisory Service (CTAS) database

Individual records in the data represent case or contact episodes, where a case corresponds to a person with a positive test for SARS-CoV-2 and no case report in the 14 days prior. A contact is a person who was named by a case as being a contact between two days before the date of the case's onset of symptoms (or test date, if not symptomatic) and the date of tracing. This includes being a member of or visitor to the case's household, or close contact during an activity or in a work or education setting. An episode is an individual entry in NHS Test and Trace based on one of the above two definitions.

Delay group

Primary case: Confirmed case with laboratory evidence of infection as determined by detection of SARS-CoV-2 RNA by nucleic acid amplification, who had no case report in the 14 days prior to their test date, and who was known from SGSS records to have been impacted by the serious untoward incident (which affected some case records from 25 September to 2 October 2020).
Primary contact: Person who was identified as a contact of a primary case during their infectious period (between two days before the date of the case's onset of symptoms or test date, if not symptomatic, and the date of tracing).
Secondary case: Person defined as a primary contact with evidence of infection as determined by detection of SARS-CoV-2 RNA by nucleic acid amplification and had a contact event with the primary case between two days and 14 days inclusive prior to the onset of symptoms (or test date if no symptom onset available) of the secondary case.
Secondary contact: Person who was identified as a contact of a secondary case during their infectious period (between two days before the date of the case's onset of symptoms or test date, if not symptomatic, and the date of tracing).
Tertiary case: Person defined as a secondary contact with evidence of infection as determined by detection of SARS-CoV-2 RNA by nucleic acid amplification and had a contact event with the primary case between two days and 14 days inclusive prior to the onset of symptoms (or test date if no symptom onset available) of the tertiary case.

Control group

Primary case: Confirmed case with laboratory evidence of infection as determined by detection of SARS-CoV-2 RNA by nucleic acid amplification, who had no case report in the 14 days prior to their test date, and who was not on the list of case records affected by the incident, but had a CTAS upload date from within the time period of the incident (25 September to 2 October 2020 inclusive).
Primary contact: Person who was identified as a contact of a primary case during their infectious period (between two days before the date of the case's onset of symptoms or test date, if not symptomatic, and the date of tracing).
Secondary case: Person defined as a primary contact with evidence of infection as determined by detection of SARS-CoV-2 RNA by nucleic acid amplification and had a contact event with the

Supplementary Figure 2: Diagram showing the different definitions for the time taken to initiate and complete contact tracing. The length of time taken for each stage in the diagram is the median time taken for that stage in the control group.

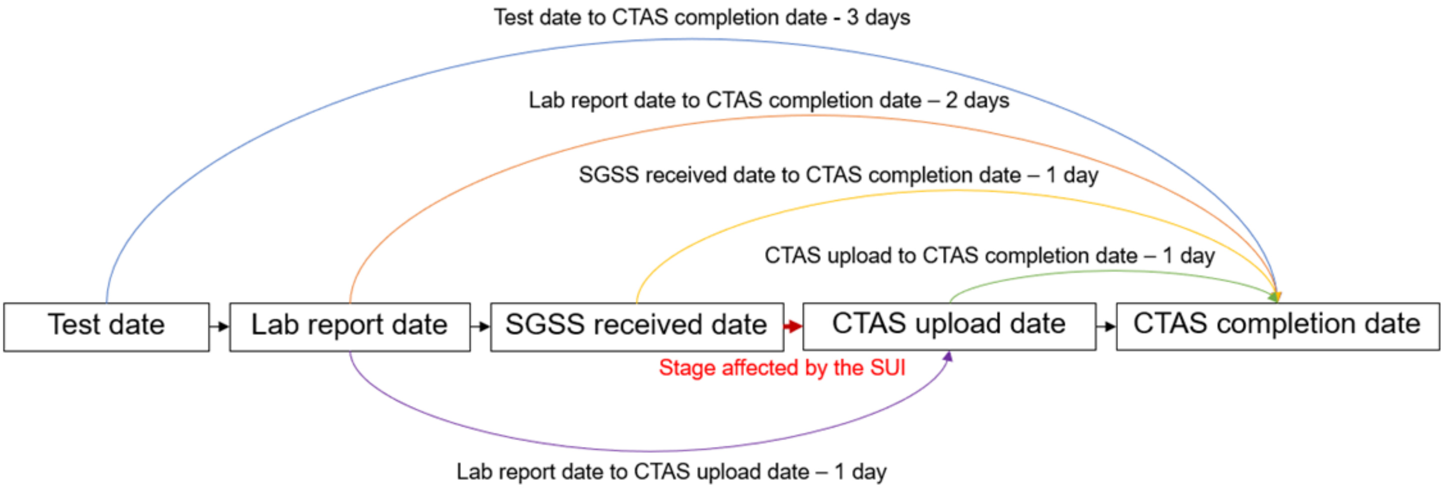

Supplementary Table 1: Description of each step of the process of matching SGSS records to CTAS records

Field(s) used for matching	Records matched (n = 15,861)
1. CDR Specimen Request SK number*	14,655
2. NHS number, forename, surname, date of birth, postcode	657
3. NHS number, forename, surname, date of birth	64
4. NHS number, date of birth, postcode	18
5. NHS number	1
6. Forename, surname, date of birth, postcode	72
**Total matched**	**15,467 (98%)**

* CDR Specimen Request SK number is the unique number identifier for each SGSS record

Supplementary Figure 3: Data flow chart describing exclusion criteria and de-duplication performed on the dataset of primary cases affected by the contact tracing delay caused by the incident. Only cases arising from community testing (pillar 2 testing) were affected; therefore, non-pillar 2 test episodes (including hospital testing) were excluded from the control group.

For peer review only

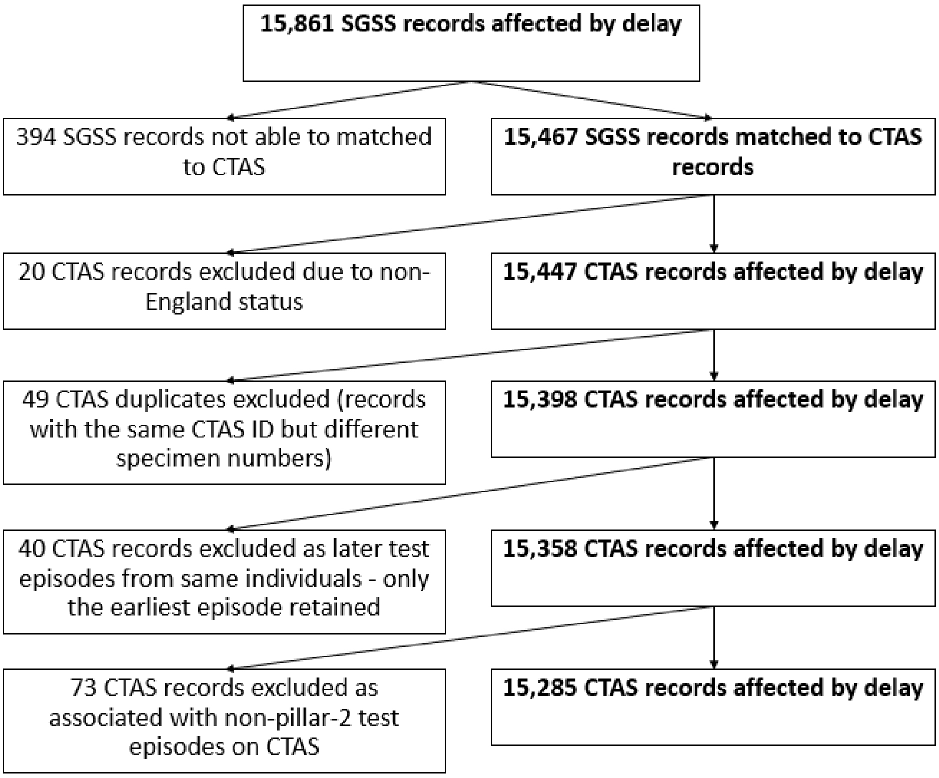
15,861 SGSS records affected by delay
394 SGSS records not able to matched to CTAS
15,467 SGSS records matched to CTAS records
20 CTAS records excluded due to non-England status
15,447 CTAS records affected by delay
49 CTAS duplicates excluded (records with the same CTAS ID but different specimen numbers)
15,398 CTAS records affected by delay
40 CTAS records excluded as later test episodes from same individuals - only the earliest episode retained
15,358 CTAS records affected by delay
73 CTAS records excluded as associated with non-pillar-2 test episodes on CTAS
15,285 CTAS records affected by delay

Supplementary Table 2: Case demographics and contact tracing outcomes

	Delay group			Control group		
**Cases**	**Primary**	**Secondary**	**Tertiary**	**Primary**	**Secondary**	**Tertiary**
Number of observations	15,285	2748	382	43,742	9575	1335
Number of individuals	15,285 (100%)	2695 (98%)	375 (98%)	43,742 (100%)	9382 (98%)	1307 (98%)
Sex						
Male	7300 (48%)	1242 (45%)	165 (43%)	20,674 (47%)	4186 (44%)	606 (45%)
Female	7985 (52%)	1436 (52%)	203 (53%)	23,067 (53%)	5105 (53%)	692 (52%)
NA	0 (0%)	70 (3%)	14 (4%)	1 (0%)	284 (3%)	37 (3%)
Age (years)						
< 18	1360 (9%)	225 (8%)	33 (9%)	3509 (8%)	881 (9%)	158 (12%)
18 – 29	6863 (45%)	1303 (47%)	198 (52%)	19,806 (45%)	4432 (46%)	651 (49%)
30 – 49	4030 (26%)	591 (22%)	75 (20%)	10,951 (25%)	2102 (22%)	257 (19%)
50 – 69	2585 (17%)	556 (20%)	70 (18%)	7800 (18%)	1852 (19%)	234 (18%)
>= 70	447 (3%)	68 (2%)	6 (2%)	1676 (4%)	298 (3%)	33 (3%)
NA	0 (0%)	5 (0%)	0 (0%)	0 (0%)	10 (0%)	2 (0%)
Ethnicity						
Black/ African/ Caribbean/ Black British	258 (2%)	30 (1%)	5 (1%)	804 (2%)	101 (1%)	6 (0%)
Indian (Asian or Asian British)	463 (3%)	90 (3%)	16 (4%)	1420 (3%)	296 (3%)	38 (3%)
Mixed/ Multiple ethnic groups	324 (2%)	51 (2%)	13 (3%)	1071 (2%)	181 (2%)	22 (2%)
Other	156 (1%)	19 (1%)	5 (1%)	402 (1%)	64 (1%)	5 (0%)
Other Asian/ Asian British	435 (3%)	87 (3%)	2 (1%)	1179 (3%)	192 (2%)	24 (2%)
Pakistani (Asian or Asian British)	702 (5%)	91 (3%)	13 (3%)	1829 (4%)	286 (3%)	19 (1%)
White	8525 (56%)	1563 (57%)	196 (51%)	26,100 (60%)	5466 (57%)	748 (56%)
Not stated	4422 (29%)	817 (30%)	132 (35%)	10,937 (25%)	2989 (31%)	473 (35%)
Geographic region						
East Midlands	1042 (7%)	202 (7%)	40 (10%)	3702 (8%)	993 (10%)	170 (13%)
East of England	373 (2%)	74 (3%)	11 (3%)	1234 (3%)	290 (3%)	55 (4%)
London	1174 (8%)	196 (7%)	20 (5%)	3966 (9%)	755 (8%)	111 (8%)
North East	1974 (13%)	338 (12%)	50 (13%)	4209 (10%)	914 (10%)	123 (9%)

For peer review only

North West	5400 (35%)	935 (34%)	110 (29%)	14,679 (34%)	3125 (33%)	372 (28%)
South East	682 (4%)	139 (5%)	23 (6%)	2385 (5%)	489 (5%)	91 (7%)
South West	481 (3%)	95 (3%)	14 (4%)	1718 (4%)	336 (4%)	55 (4%)
West Midlands	1380 (9%)	203 (7%)	27 (7%)	3707 (8%)	791 (8%)	115 (9%)
Yorkshire and the Humber	2713 (18%)	549 (20%)	86 (23%)	7876 (18%)	1837 (19%)	239 (18%)
NA	66 (0%)	17 (1%)	1 (0%)	266 (1%)	45 (1%)	4 (3%)
Completed contact tracing (n (%, 95%CI))						
Yes	12221 (80%, 79-81%)	2137 (78%, 76-79%)	276 (72%, 67-77%)	36,503 (83%, 83-84%)	7291 (76%, 75-77%)	971 (73%, 70-75%)
Difference in proportions who completed contact tracing (delay group minus control group) (95% CI)	-3.5% (-4.2% to -2.8%)	1.6% (-0.2% to 3.4%)	-0.5% (-5.7% to 4.8%)	N/A	N/A	N/A
Median (and IQR) number of contacts reported per case						
All	3 (1-4)	3 (1-4)	3 (1-5)	3 (1-4)	3 (1-4)	3 (1-4)
Household	2 (1-3)	2 (1-3)	3 (1-4)	2 (1-3)	2 (1-4)	2 (1-3)
Non-household	0 (0-1)	0 (0-0)	0 (0-0)	0 (0-1)	0 (0-0)	0 (0-0)
Mean number of contacts reported per case						
All	3.0	3.0	3.6	3.2	3.1	3.2
Household	2.2	2.4	2.6	2.3	2.5	2.4
Non-household	0.8	0.6	1.0	0.9	0.6	0.7
Proportion of cases with ≥2 household contacts (n (%, 95%CI))	7361 (48%, 47-49%)	1348 (49%, 47-51%)	182 (48%, 43-53%)	22,244 (51%, 50-51%)	4717 (49%, 48-50%)	631 (47%, 45-50%)
Median date of test	28/09/2020	01/10/2020	03/10/2020	01/10/20	03/10/2020	05/10/2020
Median (and IQR) time taken to initiate contact tracing, days between (median (IQR)):						

For peer review only

Test date to CTAS completion date	6 (4-7)	4 (2-5)	4 (2-5)	3 (2-5)	3 (2-5)	3 (2-5)
SGSS lab report date to CTAS completion date	4 (3-6)	2 (1-4)	2 (1-4)	1 (1-3)	2 (1-3)	1 (1-3)
SGSS received date to CTAS completion date	2 (1-3)	1 (1-3)	1 (1-3)	1 (1-3)	1 (1-3)	1 (1-2)
CTAS upload date to CTAS completion date	1 (1-2)	1 (0-2)	1 (0-2)	1 (0-2)	1 (0-2)	1 (0-2)
SGSS lab report date to CTAS upload date	3 (2-4)	1 (0-2)	1 (0-1)	1 (0-1)	1 (0-1)	1 (0-1)
Mean time taken for contact tracing, time between:						
SGSS lab report date to CTAS upload date	3.4	1.3	1.1	1.0	0.8	0.8
Test date to CTAS completion date	6.1	4.1	4.2	3.5	3.7	3.7

For peer review only

Supplementary Table 3: Contact demographics and contact tracing outcomes

	Delay group		Control group	
**Contacts**	**Primary**	**Secondary**	**Primary**	**Secondary**
Number of observations	37,373	6540	121,205	23,119
Number of individuals	34,047 (91%)	6045 (92%)	109,907 (91%)	21,201 (92%)
Sex				
Male	8549 (23%)	1486 (23%)	31,318 (26%)	5283 (23%)
Female	10,002 (27%)	1767 (27%)	37,269 (31%)	6260 (27%)
NA	18,822 (50%)	3287 (50%)	52,618 (43%)	11,576 (50%)
Age (years)				
< 18	3000 (8%)	394 (6%)	9989 (8%)	1653 (7%)
18 – 29	7581 (20%)	1548 (24%)	30,151 (24%)	5486 (24%)
30 – 49	4319 (12%)	695 (11%)	15,050 (12%)	2336 (10%)
50 – 69	3480 (9%)	599 (9%)	12,464 (10%)	2011 (9%)
>= 70	428 (1%)	60 (1%)	1875 (2%)	246 (1%)
NA	18,565 (50%)	3244 (50%)	51,706 (43%)	11,387 (49%)
Ethnicity				
Black/ African/ Caribbean/ Black British	360 (1%)	54 (1%)	1352 (1%)	178 (1%)
Indian (Asian or Asian British)	722 (2%)	147 (2%)	2434 (2%)	494 (2%)
Mixed/ Multiple ethnic groups	558 (1%)	90 (1%)	2111 (2%)	331 (1%)
Other	190 (1%)	30 (0%)	636 (1%)	78 (0%)
Other Asian/ Asian British	641 (2%)	124 (2%)	2209 (2%)	329 (1%)
Pakistani (Asian or Asian British)	908 (2%)	139 (2%)	3107 (3%)	428 (2%)
White	14,236 (38%)	2504 (38%)	53,468 (44%)	9122 (39%)
NA	19,758 (53%)	3452 (53%)	55,888 (46%)	12,159 (53%)
Geographic region				
East Midlands	2660 (7%)	543 (8%)	12,198 (10%)	2836 (12%)
East of England	932 (2%)	163 (2%)	3356 (3%)	697 (3%)
London	2469 (7%)	436 (6%)	9614 (8%)	1742 (8%)
North East	4949 (13%)	828 (13%)	11,818 (10%)	2284 (10%)
North West	12,753 (34%)	2150 (33%)	38,641 (32%)	7064 (31%)

For peer review only

South East	1599 (4%)	324 (5%)	6568 (5%)	1087 (5%)
South West	1194 (3%)	229 (4%)	5315 (4%)	781 (3%)
West Midlands	3405 (9%)	486 (7%)	10,175 (8%)	2072 (9%)
Yorkshire and the Humber	7293 (20%)	1348 (21%)	23,002 (19%)	4444 (19%)
NA	119 (0%)	33 (1%)	518 (0%)	112 (0%)
Completed contact tracing (n (%, 95%CI))				
Yes	19,072 (51%, 51-52%)	3361 (51%, 50-53%)	69,702 (58%, 57-58%)	11,915 (52%, 51-52%)
Difference in proportions who completed contact tracing (delay group minus control group) (95% CI)	-6.5% (-7.1% to -5.9%)	-0.1% (-1.5% to 1.2%)	N/A	N/A
Nature of contact (n (%, 95%CI))				
Household	27,477 (74%, 73-74%)	5152 (79%, 78-80%)	85,538 (71%, 70-71%)	18,221 (79%, 78-79%)
Non-household	9,635 (26%, 25-26%)	1,371 (21%, 20-22%)	34,699 (29%, 28-29%)	4609 (20%, 19-20%)
NA	261 (1%)	17 (0%)	968 (1%)	289 (1%)
Median time taken to initiate contact tracing, days between (median (IQR)):				
Test date of associated case and contact's CTAS completion date	6 (5-8)	4 (3-6)	3 (2-5)	4 (3-5)
SGSS lab report date of associated case and contact's completion date	5 (4-7)	3 (1-4)	2 (1-4)	2 (1-4)
SGSS received date of associated case and contact's completion date	3 (1-4)	2 (1-4)	2 (1-3)	2 (1-3)
CTAS upload date of case and contact's completion date	2 (1-3)	2 (1-3)	1 (0-3)	2 (1-3)

For peer review only

CTAS upload date of contact and contact's completion date	1 (0-2)	1 (0-2)	1 (0-1)	1 (0-1)
SGSS lab report date of associated case and contact's CTAS upload date	4 (3-6)	2 (1-4)	1 (1-3)	1 (1-3)
Mean time taken for contact tracing, days between				
SGSS lab report date of associated case and contact's upload date	4.5	2.4	2.0	2.0
Test date of associated case and contact's completion date	6.4	4.4	3.9	4.2
Estimated compliance of cases with self-isolation, for non-household contact events only*				
Contact event date was before test date of associated case	9276 (94%) (n = 9820)	1289 (93%) (n = 1383)	33,284 (94%) (n = 35,368)	4689 (97%) (n = 4852)
Contact event date was before laboratory report date of associated case	9553 (97%) (n = 9816)	1335 (97%) (n = 1377)	34,867 (99%) (n = 35,277)	4744 (98%) (n = 4822)
Contact event date was before symptom onset date of associated case	7245 (78%) (n = 9267)	1069 (83%) (n = 1288)	25,167 (76%) (n = 33,136)	3843 (87%) (n = 4437)

For peer review only

*The compliance of cases with self-isolation was explored by describing whether the date of test, laboratory report, and symptom onset of the case occurred before or after the date of contact with their associated contacts, for non-household contacts only. If the case made non-household contacts after taking a test, developing symptoms, or being informed they had tested positive (using laboratory report date as a proxy), this suggests a lack of compliance with self-isolation.

Supplementary Table 4: Description of the odds ratio for primary and secondary contacts of experiencing health outcomes (mortality or hospitalisation) comparing the delay group to (1) all controls in the control group and (2) to rapidly traced controls in the control group*

	**Comparison of odds in each group***
**Primary contacts**	**Crude odds ratio**
Deaths 28 day definition	
All controls	0.9 (0.4 to 1.6)
Rapidly traced controls	0.7 (0.3 to 1.3)
Deaths 60 day definition	
All controls	0.6 (0.3 to 1.1)
Rapidly traced controls	0.5 (0.2 to 1.0)
Admission to hospital as inpatient	
All controls	1.1 (1.0 to 1.2)
Rapidly traced controls	1.1 (1.0 to 1.1)
**Secondary contacts**	
Deaths 28 day definition	
All controls	0 (NA to Inf)
Rapidly traced controls	0 (NA to Inf)
Deaths 60 day definition	
All controls	0.5 (0.0 to 2.8)
Rapidly traced controls	0.5 (0.0 to 3.4)
Admission to hospital as inpatient	
All controls	1.1 (0.9 to 1.3)
Rapidly traced controls	0.9 (0.8 to 1.1)

*The control group is the reference group (odds ratio = 1), compared to the delay group. The control group consisted of either all controls, or rapidly traced controls, defined as contacts with a date of contact tracing completion within 3 days of the date of test of the associated case.

For peer review only

BMJ Open

Evaluating the impact on health outcomes of an event that resulted in a delay in contact tracing of COVID-19 cases in England, September 2020: an observational study

Journal:	*BMJ Open*
Manuscript ID	bmjopen-2022-064982.R1
Article Type:	Original research
Date Submitted by the Author:	27-Jun-2023
Complete List of Authors:	Findlater, Lucy; UK Health Security Agency South of England<br>Pierotti, Livia; University of Bristol<br>Turner, Charlie; UK Health Security Agency East of England<br>Wensley, Adrian; UK Health Security Agency North of England<br>Chen, Cong; UK Health Security Agency East of England<br>Seaman, Shaun; MRC Biostatistics Unit<br>Samartsidis, Pantelis; MRC Biostatistics Unit<br>Charlett, Andre; UK Health Security Agency<br>Anderson, Charlotte ; UK Health Security Agency<br>Hughes, Gareth; UK Health Security Agency North of England<br>Hickman, Matthew; University of Bristol<br>Edeghere, Obaghe; UK Health Security Agency Midlands and East of England<br>Oliver, Isabel; UK Health Security Agency South of England
**Primary Subject Heading**:	Public health
Secondary Subject Heading:	Epidemiology
Keywords:	COVID-19, EPIDEMIOLOGY, INFECTIOUS DISEASES, PUBLIC HEALTH

SCHOLARONE™
Manuscripts

I, the Submitting Author has the right to grant and does grant on behalf of all authors of the Work (as defined in the below author licence), an exclusive licence and/or a non-exclusive licence for contributions from authors who are: i) UK Crown employees; ii) where BMJ has agreed a CC-BY licence shall apply, and/or iii) in accordance with the terms applicable for US Federal Government officers or employees acting as part of their official duties; on a worldwide, perpetual, irrevocable, royalty-free basis to BMJ Publishing Group Ltd ("BMJ") its licensees and where the relevant Journal is co-owned by BMJ to the co-owners of the Journal, to publish the Work in this journal and any other BMJ products and to exploit all rights, as set out in our licence.

The Submitting Author accepts and understands that any supply made under these terms is made by BMJ to the Submitting Author unless you are acting as an employee on behalf of your employer or a postgraduate student of an affiliated institution which is paying any applicable article publishing charge ("APC") for Open Access articles. Where the Submitting Author wishes to make the Work available on an Open Access basis (and intends to pay the relevant APC), the terms of reuse of such Open Access shall be governed by a Creative Commons licence – details of these licences and which Creative Commons licence will apply to this Work are set out in our licence referred to above.

Other than as permitted in any relevant BMJ Author's Self Archiving Policies, I confirm this Work has not been accepted for publication elsewhere, is not being considered for publication elsewhere and does not duplicate material already published. I confirm all authors consent to publication of this Work and authorise the granting of this licence.

For peer review only

Title

Evaluating the impact on health outcomes of an event that resulted in a delay in contact tracing of COVID-19 cases in England, September 2020: an observational study

Authors

Lucy Findlater[1,2], Livia Pierotti[1,3], Charlie Turner[2], Adrian Wensley[2], Cong Chen[2], Shaun Seaman[4], Pantelis Samartsidis[4], Andre Charlett[1,2], Charlotte Anderson[2], Gareth Hughes[2], Matt Hickman[1,3], Obaghe Edeghere[2], Isabel Oliver[1,2,3]

Corresponding author: Livia Pierotti, Population Health Sciences, University of Bristol, Oakfield House, Oakfield Grove, Bristol, BS8 2BN. Email: livia.pierotti@bristol.ac.uk. Phone: 01173310128

Affiliations

[1] National Institute of Health Research Health Protection Research Unit on Behavioural Science and Evaluation at the University of Bristol, Bristol, UK
[2] UK Health Security Agency, UK
[3] Population Health Sciences, Bristol Medical School, University of Bristol, UK
[4] MRC Biostatistics Unit, University of Cambridge, East Forvie Building, Forvie Site, UK

For peer review only

Abstract

Objective*:* In September 2020, 15,861 SARS-CoV-2 case records failed to upload from the Second Generation Laboratory Surveillance System (SGSS) to the Contact Tracing Advisory Service (CTAS) tool, delaying the contact tracing of these cases. This study used CTAS data to determine the impact of this delay on population health outcomes: transmission events, hospitalisations, and mortality. Previously, a modelling study suggested a substantial impact.

Design: Observational study

Setting: England.

Population: Individuals testing positive for SARS-CoV-2 and their reported contacts.

Main outcome measures: Secondary attack rates (SARs), hospitalisations, and deaths amongst primary and secondary contacts were calculated, compared to all other concurrent, unaffected cases. Affected SGSS records were matched to CTAS records. Successive contacts and cases were identified and matched to hospital episode and mortality outcomes.

Results: Initiation of contact tracing was delayed by 3 days on average in the primary cases in the delay group (6 days) compared to the control group (3 days). This was associated with lower completion of contact tracing: 80% (95%CI: 79-81%) in delay group and 83% (95%CI: 83-84%) in control group. There was some evidence to suggest increased transmission to non-household contacts amongst those affected by the delay. The SAR for non-household contacts was higher amongst secondary contacts in the delay group than the control group (delay group: 7.9%, 95%CI: 6.5% to 9.2%; control group: 5.9%, 95%CI: 5.3% to 6.6%). There did not appear to be a significant difference between the delay and control groups in the odds of hospitalisation (crude odds ratio: 1.1 (95%CI: 0.9 to 1.2) or death (crude odds ratio: 0.7 (0.1 to 4.0)) amongst secondary contacts.

Conclusions: Our analysis suggests that the delay in contact tracing had a limited impact on population health outcomes; however, contact tracing was not completed for all individuals, so some transmission events might not be captured.

For peer review only - http://bmjopen.bmj.com/site/about/guidelines.xhtml

Strengths and limitations of the study

- Shows empirical data on the health impact of an event leading to a delay in contact tracing so can test hypotheses generated by models of the potential impact of a delay in contact tracing
- Estimates the extent of further transmission and odds of increased hospitalisation or mortality in up to the third generation of cases affected by the event
- The event acts as a natural experiment to describe the possible impact of contact tracing, comparing a group affected by chance by delayed contact tracing to a control group who experienced no delay
- Contact tracing was not completed for all individuals, so the study might not capture all affected contacts or transmissions

Introduction

As of October 2022, there have been over 22 million cases and 200,000 deaths from COVID-19 reported in the UK[1]. Contact tracing has been a central part of the public health response to SARS-CoV-2 and involves identifying contacts of people who have tested positive and advising them to self-isolate to reduce onward transmission[2]. On 28 May 2020, a national contact tracing system, NHS Test and Trace, was launched. All PCR positive cases in England were identified and contacted by phone, digital tools, and through partnerships with local authorities[3,4]. Between 25 September and 2 October 2020, some data files containing test results from community testing sites failed to import from the laboratory surveillance system (Second Generation Surveillance System (SGSS)) to the contact tracing tool (Contact Tracing and Advisory Service (CTAS)). The files contained 15, 861 new positive cases[5]. At that time, on 28 September 2020, there was a 7-day average of around 8,200 reported cases per day in England with significant regional variation[1].

The event did not affect the notification of test results to patients or affect the results of any specific testing sites, geographical areas, or population groups. However, the delay in the upload of case records to CTAS resulted in a delay in the contact tracing of these cases, and therefore a potential delay in instructing their contacts to self-isolate, which could have resulted in further transmission of SARS-CoV-2, and potentially more COVID-19 related hospitalisations and deaths. We expect that health outcomes of the initial group of cases (primary cases) whose records failed to upload to CTAS would not have been affected, as they received their test results and advice to isolate in the usual timeframe, and transmission events from primary cases to their contacts (primary contacts who may become secondary cases) would have most likely occurred prior to the isolation of the case. However, these primary contacts may not have been aware that they were a close contact of a case or advised to isolate in a timely manner, which may have increased the risk of transmission to their contacts (secondary contacts who may become tertiary cases). The outcomes of secondary contacts are therefore the most likely to have been impacted by the delay in contact tracing. In addition, there could potentially be delays in secondary cases seeking healthcare support, increasing their risk of hospitalisation or mortality.

A published preliminary model suggested that this event may have been associated with an increase of up to a third additional infections and 30 - 40% additional deaths[6].

For peer review only - http://bmjopen.bmj.com/site/about/guidelines.xhtml

In this study, we assess directly the health impact of the event, in terms of transmissions, hospitalisations, and deaths, using data on affected and concurrent cases and their contacts.

Aims and objectives

The aims of this study are to:

- Describe the nature of any contact tracing delay experienced by cases affected by the event.
- Assess the extent of further transmission, by calculating attack rates from primary cases to their contacts and from secondary cases to their contacts.
- Assess the impact of the delay on health outcomes of primary and secondary contacts, in terms of hospitalisations and deaths.

For peer review only

Methods

Data sources

The list of SGSS records affected by the event was provided by the UK Health Security Agency (UKHSA). SGSS contains demographic and diagnostic information from laboratory test reports for patients in England who test positive for SARS-CoV-2[7]. SGSS records were matched to COVID-19 testing and contact tracing records from the CTAS database to validate the cases affected by the event. CTAS records represent SARS-CoV-2 case episodes, including information on the movements of cases in their infectious period, their contacts, and demographic and clinical characteristics (Supplementary Figure 1)[8]. Matching was conducted in repeated rounds based on combinations of the following identifiers: SGSS unique identifier; NHS number; forename; surname; date of birth; and postcode. This data set of CTAS records constituted the primary cases affected by the event, described henceforth as the 'delay group'. A control group consisting of all primary cases from the same time window (30 September to 5 October 2020 inclusive) that were unaffected by the event was used as a comparison. The event affected exclusively cases arising from community testing sites and not hospital or other testing sites; therefore, primary cases in the control group were restricted to those arising from community testing[9].

Identifying cases and contacts

Contact records were linked to the case who reported them and assigned to the same group (delay or control group) as their associated case. Secondary cases were defined as (a) individuals reported as a contact by a primary case and (b) having a contact event with the primary case between 2 and 14 days inclusive prior to the onset of symptoms of the secondary case (or test date if no symptom onset available). If the secondary case was a household contact of the primary case, the date of interaction was taken to be the date of symptom onset (or test date if no symptom onset available) of the primary case. Secondary cases may be exposed to more than one primary case; therefore, only one transmission event was chosen to link a secondary case to the primary case who most likely infected them based on the following hierarchy: (1) a household transmission event was prioritised above all other types of contact event (due to the higher risk of transmission in this setting); (2) where multiple events of the same priority occurred, the most recent exposure was selected as the transmission event. Individuals who had taken multiple SARS-CoV-2 tests generated a CTAS record for each positive test result. These were identified by matching on name, date of birth, NHS number, and contact details. Only the record with the earliest test date was retained, as contact tracing should have been undertaken on receipt of the first positive result.

Linkage to health outcomes

Contact tracing records were linked to mortality data provided by UKHSA which describe death in people with a positive SARS-CoV-2 test. Matching was performed using NHS number. Two definitions of mortality consistent with SARS-CoV-2 surveillance were used: 1) death of a person with a laboratory-confirmed positive SARS-CoV-2 test and whose death was within 60 days of the first specimen date or more than 60 days after the first specimen date if COVID-19 is mentioned on the death certificate, and 2) death of a person with a laboratory-confirmed positive SARS-CoV-2 test and whose death was within 28 days of the first positive specimen date[10].

Contact datasets were also linked to the UKHSA hospital onset COVID-19 data set (extracted on 22 November 2021), which pulls from identifiable daily feeds of data on hospitalisations: Secondary Uses Services (SUS+, a national data set describing patient hospitalisations); Emergency Care Data

For peer review only - http://bmjopen.bmj.com/site/about/guidelines.xhtml

Set (ECDS, a national data set describing patient use of urgent and emergency care); and COVID-19 testing data. The data set provides one hospital spell per case if they had a hospital admission or discharge in the 14 day period before or after their first positive SARS-CoV-2 test, including people already in hospital at the time of test. Matching was performed using NHS number, date of birth, name, and sex.

Descriptive analysis

Analysis was performed in R version 1.3.1056. Investigators LF, LP, CT, AW, and CC had access to the study data. Demographic information and contact tracing outcomes were described for the case and contact data sets. Time taken for contact tracing was estimated using different dates in the contact tracing process: dates of test, laboratory report, SGSS record creation, CTAS record creation, and contact tracing completion (Supplementary Figure 2). Time taken to initiate the contact tracing process was defined as the days between the laboratory report date of the case and the date of upload of the case or contact record into CTAS. Time taken to complete contact tracing was defined as the days between the test date of the case and the date of contact tracing completion of the case or contact. Contact tracing completion was defined as the status of the individual's CTAS record being set to 'complete' by a call handler or the individual submitting an online contact tracing form.

Regression analysis

To understand the effect of the event on transmission, adjusted secondary attack rates (SARs) and adjusted odds ratios of infection with SARS-CoV-2 amongst primary and secondary contacts were estimated via post-estimation from a logistic regression model. The predictor under investigation was whether the exposing case was in the delay or the control group. After review of crude SARs stratified by contact setting (household or non-household), effect modification of the predictor (delay or control group) by the contact setting (household or non-household) was allowed for in the model. The potential confounders included in the model were age group, sex, geographic region (one of nine UKHSA-defined geographical areas) of the contact, and whether the contact completed contact tracing.

To understand the effect of the event on health outcomes, odds ratios for experiencing hospitalisation or mortality amongst primary and secondary contacts were calculated using logistic regression with Firth penalisation, comparing individuals in the delay and control groups. When individuals had contact episodes with multiple cases, the earliest episode per individual was retained. A very small number of mortality events was reported; therefore, unadjusted, crude odds ratios are presented to avoid overfitting the regression model, and the covariate balance between the delay and control groups suggests any major confounding is unlikely. As a sensitivity analysis, health outcomes of contacts in delay group were compared to a subgroup of contacts in the control group who were contact traced rapidly, defined as within three days of the test date of the associated case.

Patient and public involvement

Patients or the public were not involved in the design, or conduct, or reporting, or dissemination plans of this research.

Results

For peer review only - http://bmjopen.bmj.com/site/about/guidelines.xhtml

Sample characteristics

Overall, 15,861 SGSS records were identified as affected by the event and 15,467 (98%) were matched to a CTAS record (Supplementary Table 1). Following data cleaning, 15,285 (96%) primary cases affected by the event remained eligible for inclusion in the study (Supplementary Figure 3). A control group of 43,742 concurrent primary cases was created, consisting of all CTAS records from the time period of 30 September to 5 October 2020 inclusive which were not affected by the event. Secondary and tertiary cases and primary and secondary contacts were identified in the CTAS database. Demographic information is described in the supplementary material (Supplementary Tables 2 and 3).

Nature of contact tracing delay

For primary cases in the delay group, it took on average two days longer for their records to be uploaded to CTAS, initiating contact tracing, after the laboratory report date of their positive test result (Table 1, Figure 1). Primary cases took on average three days longer to complete contact tracing in the delay group (6 days, interquartile range (IQR): 4-7) than the control group (3 days, (IQR: 2-5)) after their date of positive test. For primary contacts in the delay group, it took on average three days longer for their records to be uploaded to CTAS, relative to the laboratory report date of their associated case. It took three days longer to complete contact tracing for primary contacts in the delay group (6 days (IQR: 5-8)) than the control group (3 days (IQR: 2-5)), relative to the test date of their associated case (Table 2, Figure 1). Once records were uploaded onto CTAS, the primary cases and contacts in the delay group took the same median amount of time to complete contact tracing as the control group (1 day).

The proportion of cases for whom contact tracing was completed was slightly lower for primary cases in the delay group (80%, 95%CI: 79-81%) than the control group (83%, 95%CI: 83-84%), a difference of -3.5% (95%CI: -4.2% to -2.8%) (Table 1). There was no difference in the proportion of secondary and tertiary cases who completed contact tracing. The proportion of primary contacts traced was lower by 6.5% (95%CI: -7.1% to -5.9%) in the delay group than the control group (delay group 51%, 95%CI: 51-52%; control group 58%, 95%CI: 57-58%). There was no evidence of a difference for secondary contacts (delay group 51%, 95%CI: 50-53%; control group 52%, 95%CI: 51-52%) (Table 2). For primary and secondary cases, the median numbers of household and non-household contacts per case was the same in the delay and control groups; for tertiary cases, the median number was the same for non-household contacts but was greater by one person for household contacts in the delay compared to the control group (Table 1). For contacts, the nature of contact was reported as household contact for 71-79% of contacts in each group (Table 2).

For peer review only - http://bmjopen.bmj.com/site/about/guidelines.xhtml

Table 1: Contact tracing outcomes for primary, secondary, and tertiary cases

	Delay group			Control group		
**Cases**	**Primary**	**Secondary**	**Tertiary**	**Primary**	**Secondary**	**Tertiary**
Number of observations	15,285	2748	382	43,742	9575	1335
Number of individuals	15,285 (100.0%)	2695 (98.1%)	375 (98.2%)	43,742 (100.0%)	9382 (98.0%)	1307 (97.9%)
Completed contact tracing (n (%, 95%CI))						
Yes	12,221 (80.0%, 79.3%-81%)	2137 (77.8%, 76.2%-79.3%)	276 (72.3%, 67.5%-76.7%)	36,503 (83.5%, 83.1%-83.8%)	7291 (76.1%, 75.3%-77.0%)	971 (72.7%, 70.3%-75.1%)
Difference in proportions who completed contact tracing (delay group minus control group) (95% CI)	-3.5% (-4.2% to -2.8%)	1.6% (-0.2% to 3.4%)	-0.5% (-5.7% to 4.8%)	N/A	N/A	N/A
Median (and IQR) number of contacts reported per case						
All	3 (1-4)	3 (1-4)	3 (1-5)	3 (1-4)	3 (1-4)	3 (1-4)
Household	2 (1-3)	2 (1-3)	3 (1-4)	2 (1-3)	2 (1-4)	2 (1-3)
Non-household	0 (0-1)	0 (0-0)	0 (0-0)	0 (0-1)	0 (0-0)	0 (0-0)
Mean number of contacts reported per case						
All	3.0	3.0	3.6	3.2	3.1	3.2
Household	2.2	2.4	2.6	2.3	2.5	2.4
Non-household	0.8	0.6	1.0	0.9	0.6	0.7
Number of cases with ≥2 household contacts (n (%, 95%CI))	7361 (48.2%, 47.4%-49.0%)	1348 (49.1%, 47.2%-50.9%)	182 (47.6%, 42.5%-52.8%)	22,244 (50.9%, 50.4%-51.3%)	4717 (49.3%, 48.3%-50.3%)	631 (47.3%, 44.6%-50.0%)
Median (and IQR) time taken for contact tracing, days between:						
SGSS lab report date to CTAS upload date	3 (2-4)	1 (0-2)	1 (0-1)	1 (0-1)	1 (0-1)	1 (0-1)
Test date to CTAS completion date	6 (4-7)	4 (2-5)	4 (2-5)	3 (2-5)	3 (2-5)	3 (2-5)

For peer review only - http://bmjopen.bmj.com/site/about/guidelines.xhtml

Mean time taken for contact tracing, time between:						
SGSS lab report date to CTAS upload date	3.4	1.3	1.1	1.0	0.8	0.8
Test date to CTAS completion date	6.1	4.1	4.2	3.5	3.7	3.7

Table 2: Contact tracing outcomes for primary and secondary contacts

	**Delay group**		**Control group**	
**Contacts**	**Primary**	**Secondary**	**Primary**	**Secondary**
Number of observations	37,373	6540	121,205	23,119
Number of individuals	34,047 (91.1%)	6045 (92.4%)	109,907 (90.7%)	21,201 (91.7%)
Completed contact tracing (n (%, 95%CI))				
Yes	19,072 (51.0%, 50.5%-51.5%)	3361 (51.4%, 50.2%-52.6%)	69,702 (57.5%, 57.2%-57.8%)	11,915 (51.5%, 50.9%-52.2%)
Difference in proportions who completed contact tracing (delay group minus control group) (95% CI)	-6.5% (-7.1% to -5.9%)	-0.1% (-1.5% to 1.2%)	N/A	N/A
Nature of contact (n (%, 95%CI))				
Household	27,477 (73.5%, 73.1%-74.0%)	5152 (78.8%, 77.8%-79.8%)	85,538 (70.6%, 70.3%-70.8%)	18,221 (78.8%, 78.3%-79.3%)
Non-household	9,635 (25.8%, 25.3%-26.2%)	1371 (21.0%, 20.0%-22.0%)	34,699 (28.6%, 28.4%-28.9%)	4609 (19.9%, 19.4%-20.5%)
NA	261 (0.7%)	17 (0.3%)	968 (0.8%)	289 (1.3%)
Median (and IQR) time taken for contact tracing, days between:				
SGSS lab report date of associated case and contact's upload date	4 (3-6)	2 (1-4)	1 (1-3)	1 (1-3)
Test date of associated case and	6 (5-8)	4 (3-6)	3 (2-5)	4 (3-5)

For peer review only

contact's completion date				
Mean time taken for contact tracing, days between:				
SGSS lab report date of associated case and contact's upload date	4.5	2.4	2.0	2.0
Test date of associated case and contact's completion date	6.4	4.4	3.9	4.2

For peer review only

For peer review only - http://bmjopen.bmj.com/site/about/guidelines.xhtml

Effect on transmission

There was no evidence of a difference between the delay and control groups in the overall adjusted secondary attack rates (SARs) amongst primary or secondary contacts (Table 3). However, differences were observed when stratified by household or non-household setting; SARs in non-household secondary contacts were higher for the delay group (7.9%, 95% CI 6.5% to 9.2%) than the control group (5.9%, 95% CI 5.3% to 6.6%), corresponding to 1.36 (1.09 to 1.69) times greater odds of infection. No difference was observed between the groups amongst household secondary contacts. For primary contacts, household SARs were lower for the delay group than for the control group. Conversely, for non-household contacts, the SARs in primary contacts were higher for the delay group (6.3%, 95% CI 5.8% to 6.8%) than for the control group (5.7%, 95% CI 5.5% to 5.9%), corresponding to 1.12 (1.02 to 1.22) greater odds of infection.

Effect on health outcomes

COVID-19 death was a rare outcome in both groups with no evidence of a difference in 28 or 60 day mortality rates amongst primary or secondary contacts in the delay and control groups (Table 4). Using the 28 day definition of mortality, for primary contacts, there were 2.9 deaths (95%CI: 1.4 – 5.4) per 10,000 in the delay group compared to 3.7 deaths (95%CI: 2.7 – 5.1) per 10,000 in the control group. For secondary contacts, this was 0.0 (95%CI: 0.0 – 6.1) deaths per 10,000 in the delay group compared to 1.4 (95%CI: 0.3 – 4.1) deaths per 10,000 in the control group. There was no evidence for a difference in the odds of mortality for the delay group compared to the control group (Table 4, Figure 2).

The proportion of contacts admitted to hospital as inpatients within 14 days of their first SARS-CoV-2 test was almost identical in the delay and control groups, for primary contacts (delay 3.3% (95%CI: 3.1-3.5%), control 3.1% (95%CI: 3.0-3.2%)) and secondary contacts (delay 3.4% (95%CI: 3.0-3.9%), control 3.2% (95%CI: 3.0-3.5%)) (Table 4). There was no significant difference in the crude or adjusted odds of hospital admission between the delay and control groups (Table 4, Figure 2). Additionally, there was no significant difference in the crude or adjusted odds of hospitalisation or death when the delay group was compared to a subset of the control group who were contact traced most rapidly (Supplementary Table 4).

For peer review only - http://bmjopen.bmj.com/site/about/guidelines.xhtml

Table 3: Adjusted secondary attack rates and odds ratios of infection amongst contacts of primary and secondary cases in the delay group compared to the control group, by setting of contact

Setting of contact event	Delay			Control			Adjusted* odds ratio of infection (95% CI)**
	Number of contacts	Contacts becoming cases	Adjusted* secondary attack rate (SAR) (95% CI)	Number of contacts	Contacts becoming cases	Adjusted* secondary attack rate (SAR) (95% CI)	
**Primary contacts**							
All	37,373	2,702	7.5% (7.3 to 7.8%)	121,205	9,419	7.7% (7.5 to 7.8%)	0.99 (0.94 to 1.03)
Household	27,477	2,063	8.1% (7.8 to 8.4%)	85,538	7,195	8.6% (8.4 to 8.7%)	0.94 (0.89 to 0.99)
Non-household	9,896	639	6.3% (5.8 to 6.8%)	35,667	2,224	5.7% (5.5 to 5.9%)	1.12 (1.02 to 1.22)
**Secondary contacts**							
All	6,540	377	5.8% (5.2 to 6.3%)	23,119	1,315	5.7% (5.4 to 6.0%)	0.99 (0.88 to 1.12)
Household	5,152	256	5.1% (4.5 to 5.7%)	18,221	990	5.6% (5.3 to 6.0%)	0.91 (0.79 to 1.05)
Non-household	1,388	121	7.9% (6.5% to 9.2%)	4,898	325	5.9% (5.3 to 6.6%)	1.36 (1.09 to 1.69)

*Adjusted SARs and odds ratios allowing for effect modification of the study type by household, and adjusted for the age group, sex and geographic region of the contact, and whether they completed contact tracing

**Adjusted odds ratio of infection, comparing the odds of SARS-CoV-2 infection amongst contacts in the control group (reference group) to the delay group

Table 4: Odds ratios for mortality and hospital outcomes in the delay group compared to the control group

	Delay group		Control group		
**Primary contacts**	**Number (n = 34,047)**	**Proportion**	**Number (n = 109,907)**	**Proportion**	**Crude odds ratio***
Deaths 28 day definition	10	2.9 (1.4 – 5.4) per 10,000 population	41	3.7 (2.7 – 5.1) per 10,000 population	0.8 (0.4 to 1.6)
Deaths 60 day definition	10	2.9 (1.4 – 5.4) per 10,000 population	61	5.6 (4.2 – 7.1) per 10,000 population	0.6 (0.3 to 1.1)

For peer review only - http://bmjopen.bmj.com/site/about/guidelines.xhtml

Admission to hospital as inpatient	1121	3.3% (3.1% – 3.5%)	3364	3.1% (3.0% - 3.2%)	1.1 (1.0 to 1.2)
**Secondary contacts**	**Number (n = 6045)**	**Proportion**	**Number (n = 21,201)**	**Proportion**	**Crude odds ratio***
Deaths 28 day definition	0	0.0 (0.0 – 6.1) per 10,000 population	3	1.4 (0.3 – 4.1) per 10,000 population	0.5 (0.0 to 9.7)
Deaths 60 day definition	1	1.7 (0.0 – 9.2) per 10,000 population	7	3.3 (1.3 – 6.8) per 10,000 population	0.7 (0.1 to 4.0)
Admission to hospital as inpatient	206	3.4% (3.0% – 3.9%)	687	3.2% (3.0% – 3.5%)	1.1 (0.9 to 1.2)

*The control group is the reference group (odds ratio = 1)

For peer review only

Discussion

Statement of principal findings

The contact tracing of 15,861 SARS-CoV-2 cases affected by the event was delayed by an average of three days. There was evidence that the delay was associated with a slight decrease in the proportion of cases and contacts who completed contact tracing. Despite the delay, the mean and median number of overall contacts and non-household contacts made per case was similar in the delay and control groups, for primary, secondary, and tertiary cases, and the median number of household contacts per case was near-identical.

As expected, we observed a higher SAR from secondary to tertiary cases, for non-household contact events in the delay group. There was no evidence of a difference in the SARs or odds of infection for secondary to tertiary cases overall or for household contact events. We found no evidence of a difference in hospitalisation or mortality among primary or secondary contacts in the delay or control group using the standard definitions for COVID-19-related hospitalisation and mortality used for surveillance purposes in the UK.

Strengths and weaknesses

The event acted as a natural experiment whereby one group of individuals by chance experienced delayed contact tracing, enabling a comparison of their outcomes with concurrent, unaffected cases and contacts to assess the impact of contact tracing more generally. Having a a single national system collating all test results and a single national contact tracing system facilitated the study. Through record linkage we could identify successive generations of contacts and cases and describe key health outcomes associated with the delay in contact tracing; however, some outcomes were rare and it is possible that we did not have the power to detect a small difference in hospitalisation or mortality.

Secondary transmission could only be estimated using contacts reported by cases who met the contact definition[11]. These would not include unknown contacts; however, the similarity in the number of reported contacts between the two groups suggests that unknown contacts are also likely to be similar. Contact tracing was not completed for a minority of cases in the delay and control groups, so there are likely to be further transmission events that are unknown and not described, and subsequent underreporting of contacts. People who do not engage in contact tracing differ from those who do in terms of ethnicity and socioeconomic status; however, this is unlikely to differ between delay and control groups[12,13,14]. Completion of contact tracing was slightly lower in the delay group; for primary contacts, this was 51% in the delay group and 58% in the control group. This could have potentially increased the likelihood of worse outcomes in this group.

It is important to note that there was an inherent lag in the initiation of contact tracing for all cases; therefore, the delay group was not compared to a perfect example of contact tracing, which may explain the lack of difference in observed health outcomes. To assess this, we conducted sensitivity analyses comparing the delay group to a subset of the control group who initiated contact tracing most quickly, but we still did not detect a difference in health outcomes and results were consistent with the main analysis. This could be partly because secondary

For peer review only - http://bmjopen.bmj.com/site/about/guidelines.xhtml

and tertiary contacts in the delay group did not appear to experience a significant delay in initiating or completing tracing compared to the control group. Additionally, some cases had multiple CTAS records, for example, if they tested several times; we retained only the earliest episode. It is possible that some cases in the delay group reported subsequent positive tests soon after their first test which were not affected by the event (for example, after being admitted to hospital). Therefore, these people might still have been contact traced in a timely manner.

Finally, there is likely to be high uncertainty when tracing the complex network of contacts from the community transmission stage of the pandemic. The actual risk of infection amongst contacts is likely to vary significantly depending on degree of contact, which was not described. Some transmissions were likely yielded by superspreading events with a disproportionate impact on the pandemic, which were not captured in our comparison of secondary attack rates. Notably, primary cases were defined as being either affected by the event (delay group) or from the same time period (control group). Because the event occurred over a period of a small number of days, during which time transmission events occurred, primary cases generated some secondary and tertiary cases which were also classed as primary cases themselves and may have been directly affected by the event. Therefore, transmission chains may have been affected at different stages. Additionally, with regard to household transmission chains, it is worth noting that because many secondary cases were exposed to the virus in their household, their onward household attack rates could be lowered as their exposer would not become a case again within the time period studied.

Strengths and weaknesses in relation to other studies

A previous modelling exercise using aggregate published data projected substantial adverse health outcomes, describing a drastic rise in SARS-CoV-2 infections and deaths in areas most affected by the event and estimating over 125,000 additional infections and 1,500 additional COVID-19-related deaths[6]. Their rapid modelling approach described broader regional trends in infections and deaths using publicly available population surveillance data from the nearest calendar week to the event. This has the advantage of capturing all individuals possibly affected by the event, including any unknown or unreported contacts. However, it could lead to an overestimation of the health impact of the event. Our study described outcomes of the cases known to be affected by the event, which were a subset of the cases from 25 September – 2 October 2020, but the previous modelling study defined affected cases as all those with specimen dates from 20 – 27 September 2020; this might also lead to less accurate estimation of health impacts. It is also important to note that the event occurred at a point in the pandemic when incidence of COVID-19 was increasing nationally and more rapidly in some regions[15,16].

Meaning of the study

Contacts of SARS-CoV-2 cases are at increased risk of infection, and this risk is higher among household contacts[17,18,19]. It is also known that significant numbers of infected people are asymptomatic and can transmit the virus[17,18,20]. Therefore, tracing all contacts of cases of SARS-CoV-2 to inform them of their exposure and give them advice on measures to reduce the risk of onward transmission, has been a cornerstone of the pandemic response. This event

For peer review only - http://bmjopen.bmj.com/site/about/guidelines.xhtml

offered an opportunity to assess the impact of contact tracing on health outcomes. Interestingly, few differences in health outcomes were observed between people impacted by the delay and concurrent cases and contacts.

There was no difference in the number of contact events. This may reflect that people have generally maintained low levels of contacts throughout the pandemic[20]..We did observe an increase in secondary transmission among non-household contacts, which is to be expected if people are not aware of their exposure and therefore not isolating. However, non-household contacts comprised a minority of reported contacts. Household contacts made up the majority of transmission events and did not experience greater secondary transmission, perhaps due to the more limited effect of self-isolation in preventing transmission within households. This could explain in part why we did not see such a large effect on transmission and health outcomes as predicted by other research.

Unanswered questions and future research

Contact tracing is an effective tool to control transmission of infection[21,22,23]. Modelling studies have estimated the benefit from contact tracing and self-isolation to reduce SARS-CoV-2 transmission[24,25,26]. There is more limited evidence on the actual impact on transmission of the very large scale contact tracing undertaken during the pandemic. For contact tracing to be effective it needs to be timely and reach, as far as possible, all contacts[27,28,29]. The lack of a significant adverse health impact observed in our study could reflect the fact that there was an inherent delay in the tracing of all cases, which has been shown to reduce the effectiveness of SARS-CoV-2 contact tracing[30,31]. The lack of a difference may also reflect that, at that time, people generally were limiting their contact with others, with low numbers of non-household contacts made by individuals in both groups[20]. Other factors that may have contributed include cases informing their contacts directly of their exposure status and need for self-isolation prior to official notification from the contact tracing system, and that a majority of contact events occurred in the household, where self-isolation is less likely to prevent transmission. All of these factors suggest that large scale contact tracing may have had a limited impact on SARS-CoV-2 transmission at that time. Future research should evaluate the most effective approaches to conducting large scale contact tracing.

Conclusions

We did not find a significant impact on population health outcomes in individuals affected by a delay in contact tracing.

Acknowledgements

We acknowledge the UK Health Security Agency (UKHSA) for permitting and facilitating access to data. We acknowledge Anne-Marie O'Connell, Richard Dunn, Tom Clare, Fernando Capelastegui, Russell Hope, Simon Collin, and Efejiro Ashano (UKHSA) and James Thomas (NHS Digital) for assisting with data acquisition and linkage. We acknowledge Daniela De Angelis (MRC Biostatistics Unit, UKHSA) for contributing to project development and feedback. We acknowledge Oliver McManus (UKHSA) for assisting with code review.

For peer review only - http://bmjopen.bmj.com/site/about/guidelines.xhtml

Contributors

LF, LP, and IO contributed to conception and design of the work, analysis and interpretation of data, and drafted the manuscript. CT, AW, CC, CA, and GH contributed to design of the work, and acquisition, analysis, and interpretation of data. SS, PS, MH, OE, and AC contributed to design of the work and interpretation of data. All authors revised the manuscript criticially and approved the final version of the manuscript.

Funding

The authors have not declared a specific grant for this research from any funding agency in the public, commercial or not-for-profit sectors. LF, LP, AC, MH, and IO acknowledge support from the NIHR Health Protection Research Unit in Behavioural Science and Evaluation at University of Bristol. PS was funded by the NIHR Programme Grants for Applied Research programme (grant RP-PG-0616-20008). SS was funded by UKRI (grant MC_UU_00002/10) and UKHSA. For the purpose of open access, the author has applied a Creative Commons Attribution (CC BY) licence to any Author Accepted Manuscript version arising.

Disclaimer

The views expressed are those of the author and not necessarily those of the NIHR, the Department of Health and Social Care, or the UKHSA.

Competing interests

No competing interests to declare.

Patient consent for publication

Not required

Ethics approval

Approval for the study was granted from the Public Health England (PHE) (now UKHSA) Research Ethics and Governance Group (REGG). R&D reference: R&D 431. Permission was obtained from UKHSA to access contact tracing, hospitalisation, and mortality data. Individual consent was not required as the study presented anonymised, population-level data.

Data availability statement

The data that support this study were collected as part of a public health response and are considered sensitive and not made publicly available. Reasonable requests for access to anonymised data and data dictionary will be considered by the authors on request.

Figure legends

Figure 1: Graphs describing the time taken to initiate and complete contact tracing of cases and contacts in the delay and control groups

Figure 2: Graph describing odds ratios for mortality and hospitalisation, comparing the delay group to the control group (reference odds ratio = 1)

For peer review only - http://bmjopen.bmj.com/site/about/guidelines.xhtml

For peer review only

For peer review only

For peer review only - http://bmjopen.bmj.com/site/about/guidelines.xhtml

References

[1] UK Government (2021). Coronavirus (COVID-19) in the UK. UK summary. Available from https://coronavirus.data.gov.uk/. Accessed on 25.04.22
[2] Public Health England (2021) NHS Test and Trace Available from https://contact-tracing.phe.gov.uk/. Accessed on 01.12.21
[3] Public Health England (2020) Letter from Richard Gleave, Deputy Chief Executive of Public Health England, describing the use of CTAS in the COVID-19 response. Available from https://assets.publishing.service.gov.uk/government/uploads/system/uploads/attachment_data/file/881231/Letter_to_DsPH_on_contact_tracing.pdf. Accessed on 01.12.21
[4] Keeling et al. (2020) Efficacy of contact tracing for the containment of the 2019 novel coronavirus (COVID-19). J Epidemiol Community Health. 2020;74:861-866.
[5] Public Health England (2020). PHE statement on delayed reporting of COVID-19 cases. Available from https://www.gov.uk/government/news/phe-statement-on-delayed-reporting-of-covid-19-cases. Accessed on 01.12.21
[6] Fetzer and Graeber (2020). Does contact tracing work? Quasi-experimental evidence from an Excel error in England. CAGE working paper no. 521. Available from https://www.pnas.org/content/118/33/e2100814118. Accessed on 01.12.21
[7] NHS Digital (2021). SGSS and CHESS data. Available from: https://digital.nhs.uk/about-nhs-digital/corporate-information-and-documents/directions-and-data-provision-notices/data-provision-notices-dpns/sgss-and-chess-data. Accessed on 01.12.21
[8] UK Health Security Agency (2022). NHS Test and Trace in the workplace. Available from: https://www.gov.uk/guidance/nhs-test-and-trace-workplace-guidance. Accessed on 04.02.22
[9] Department of Health & Social Care (2020). COVID-19 testing data: methodology note. Available from https://www.gov.uk/government/publications/coronavirus-covid-19-testing-data-methodology/covid-19-testing-data-methodology-note. Accessed on 01.12.21
[10] Public Health England (2020). PHE data series on deaths in people with COVID-19: technical summary. Available from https://www.gov.uk/government/publications/phe-data-series-on-deaths-in-people-with-covid-19-technical-summary. Accessed on 01.12.21
[11] UK Health Security Agency (2022). (Withdrawn) Guidance for contacts of people with confirmed coronavirus (COVID-19) infection who do not live with the person. Available from: https://www.gov.uk/government/publications/guidance-for-contacts-of-people-with-possible-or-confirmed-coronavirus-covid-19-infection-who-do-not-live-with-the-person/guidance-for-contacts-of-people-with-possible-or-confirmed-coronavirus-covid-19-infection-who-do-not-live-with-the-person. Accessed on 10.05.22
[12] Shelby et al. (2021). Lessons learned from COVID-19 contact tracing during a public health emergency: a prospective implementation study. Frontiers in Public Health. 2021:9. doi: 10.3389/fpubh.2021.721952
[13] Munzert S, Selb P, Gohdes A, et al. Tracking and promoting the usage of a COVID-19 contact tracing app. Nat Hum Behav 2021;5(2):247-55. doi: 10.1038/s41562-020-01044-x
[14] Pegollo et al. (2021). Characteristics and determinants of population acceptance of COVID-19 digital contact tracing: a systematic review. Acta Biomedica. 2021;92(6):e2021444. doi: 10.23750/abm.v92iS6.12234
[15] Office for National Statistics (2020). Coronavirus (COVID-19) Infection Survey pilot: England, Wales and Northern Ireland, 2 October 2020. Available from: https://www.ons.gov.uk/peoplepopulationandcommunity/healthandsocialcare/conditionsanddiseases/bulletins/coronaviruscovid19infectionsurveypilot/englandwalesandnorthernireland2october2020. Accessed on 11.05.21.
[16] Office for National Statistics (2020). Coronavirus (COVID-19) Infection Survey pilot: England, Wales and Northern Ireland, 9 October 2020. Available from: https://www.ons.gov.uk/peoplepopulationandcommunity/healthandsocialcare/conditionsanddiseases/bulletins/coronaviruscovid19infectionsurveypilot/englandwalesandnorthernireland9october2020. Accessed on 11.05.21.
[17] Marchant et al. (2021). Determining the acceptability of testing contacts of confirmed COVID-19 cases to improve secondary case ascertainment. J Public Health (Oxf). 2021;43(3):446-452. doi: 10.1093/pubmed/fdab079
[18] Love et al. (2021). The acceptability of testing contacts of confirmed COVID-19 cases using serial, self-administered lateral flow devices as an alternative to self-isolation. medRxiv. doi: https://doi.org/10.1101/2021.03.23.21254168
[19] UK Health Security Agency (2021). Insights on transmission of COVID-19 with a focus on the hospitality, retail and leisure sector. Available from: https://assets.publishing.service.gov.uk/government/uploads/system/uploads/attachment_data/file/982865/S1194_Transmission_in_hospitality_retail_leisure.pdf. Accessed on 01.12.21.

For peer review only - http://bmjopen.bmj.com/site/about/guidelines.xhtml

[20] UK Health Security Agency (2021). Weekly statistics for NHS Test and Trace (England): 14 October to 20 October 2021. Available from: https://assets.publishing.service.gov.uk/government/uploads/system/uploads/attachment_data/file/1029536/test-and-trace-oct-14-to-20.pdf. Accessed on 01.12.21.
[21] Hossain et al. (2022). Effectiveness of contact tracing in the control of infectious diseases: a systematic review. The Lancet Public Health. 2022;7(3):e259-e273
[22] Bicher et al. (2021). Evaluation of Contact-Tracing Policies against the Spread of SARS-CoV-2 in Austria: An Agent-Based Simulation. Med Decis Making. 2021;41(8):1017-1032. doi: 10.1177/0272989X2110133062
[23] Fields et al. (2021). Coronavirus Disease Contact Tracing Outcomes and Cost, Salt Lake County, Utah, USA, March–May 2020. Emerging Infectious Diseases. 27(12):2999-3008. doi: https://doi.org/10.3201/eid2712.210505
[24] Kucharski et al (2020). Effectiveness of isolation, testing, contact tracing, and physical distancing on reducing transmission of SARS-CoV-2 in different settings: a mathematical modelling study. Lancet Infect Dis . 2020;20(10):1151-1160. doi: 10.1016/S1473-3099(20)30457-6.
[25] Chen and Huang (2022). Modelling the impacts of contact tracing on an epidemic with asymptomatic infection. Applied Mathematics and Computation. 2022;416. doi: https://doi.org/10.1016/j.amc.2021.126754
[26] Amaku et al (2021). Modelling the impact of contact tracing of symptomatic individuals on the COVID-19 epidemic. Clinics (Sao Paulo). 2021;76:e2639. doi: 10.6061/clinics/2021/e2639. eCollection 2021nh
[27] Ferretti et al. (2020). Quantifying SARS-CoV-2 transmission suggests epidemic control with digital contact tracing. Science. 2020;368(6491):eabb6936. doi: 10.1126/science.abb6936. Epub 2020 Mar 31.
[28] Grantz et al. (2021). Maximizing and evaluating the impact of test-trace-isolate programs: A modelling study. PLOS Medicine. 2021;18(4): e1003585. doi: https://doi.org/10.1371/journal.pmed.1003585
[29] Ashcroft P, Lehtinen S, Bonhoeffer S. Test-trace-isolate-quarantine (TTIQ) intervention strategies after symptomatic COVID-19 case identification. PloS one 2022;17(2):e0263597. doi: 10.1371/journal.pone.0263597
[30] Soldano, G.J., et al. (2021) COVID-19 mitigation by digital contact tracing and contact prevention (app-based social exposure warnings). Sci Rep 11, 2021;14421. https://doi.org/10.1038/s41598-021-93538-5
[31] Gardner BJ and Kilpatrick AM (2021) Contact tracing efficiency, transmission heterogeneity, and accelerating COVID-19 epidemics. PLoS Comput Biol 2021;17(6): e1009122. https://doi.org/10.1371/journal.pcbi.1009122

For peer review only - http://bmjopen.bmj.com/site/about/guidelines.xhtml

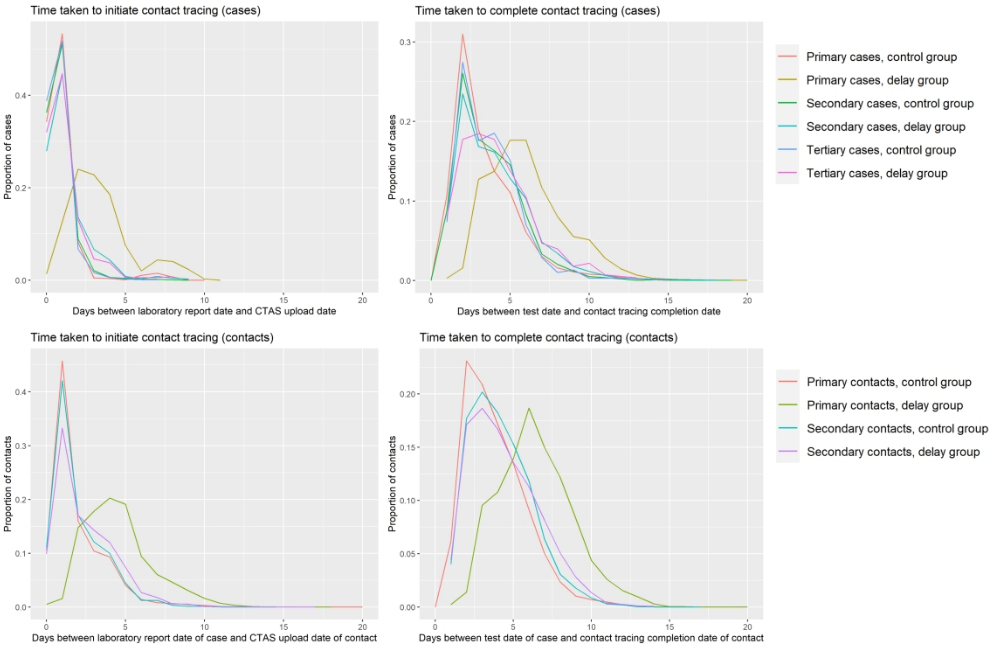

Figure 1: Graphs describing the time taken to initiate and complete contact tracing of cases and contacts in the delay and control groups

289x190mm (300 x 300 DPI)

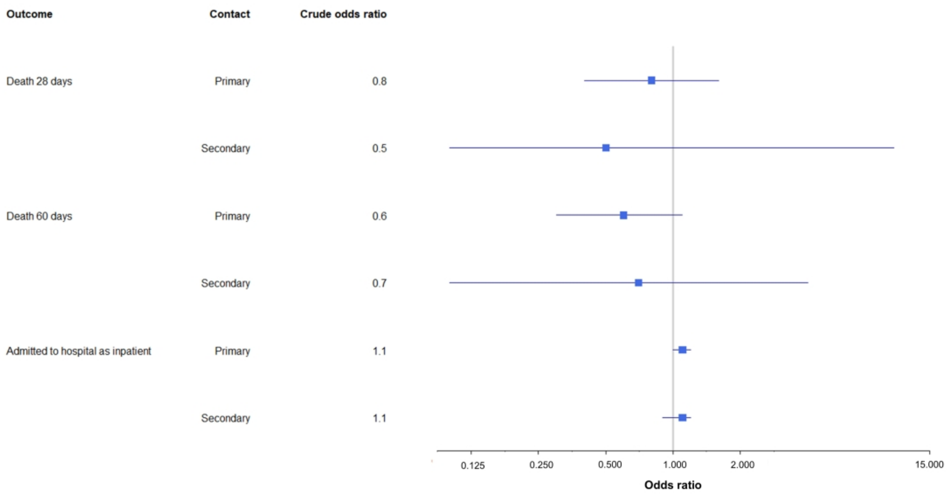

Figure 2: Graph describing odds ratios for mortality and hospitalisation, comparing the delay group to the control group (reference odds ratio = 1)

338x190mm (300 x 300 DPI)

Supplementary Material

Supplementary Figure 1: Definitions of SARS-CoV-2 cases and contacts

Definitions of cases and contacts in the Contact Tracing Advisory Service (CTAS) database
Individual records in the data represent case or contact episodes, where a case corresponds to a person with a positive test for SARS-CoV-2 and no case report in the 14 days prior. A contact is a person who was named by a case as being a contact between two days before the date of the case's onset of symptoms (or test date, if not symptomatic) and the date of tracing. This includes being a member of or visitor to the case's household, or close contact during an activity or in a work or education setting. An episode is an individual entry in NHS Test and Trace based on one of the above two definitions.

Delay group
Primary case: Confirmed case with laboratory evidence of infection as determined by detection of SARS-CoV-2 RNA by nucleic acid amplification, who had no case report in the 14 days prior to their test date, and who was known from SGSS records to have been impacted by the serious untoward incident (which affected some case records from 25 September to 2 October 2020).
Primary contact: Person who was identified as a contact of a primary case during their infectious period (between two days before the date of the case's onset of symptoms or test date, if not symptomatic, and the date of tracing).
Secondary case: Person defined as a primary contact with evidence of infection as determined by detection of SARS-CoV-2 RNA by nucleic acid amplification and had a contact event with the primary case between two days and 14 days inclusive prior to the onset of symptoms (or test date if no symptom onset available) of the secondary case.
Secondary contact: Person who was identified as a contact of a secondary case during their infectious period (between two days before the date of the case's onset of symptoms or test date, if not symptomatic, and the date of tracing).
Tertiary case: Person defined as a secondary contact with evidence of infection as determined by detection of SARS-CoV-2 RNA by nucleic acid amplification and had a contact event with the primary case between two days and 14 days inclusive prior to the onset of symptoms (or test date if no symptom onset available) of the tertiary case.

Control group
Primary case: Confirmed case with laboratory evidence of infection as determined by detection of SARS-CoV-2 RNA by nucleic acid amplification, who had no case report in the 14 days prior to their test date, and who was not on the list of case records affected by the incident, but had a CTAS upload date from within the time period of the incident (25 September to 2 October 2020 inclusive).
Primary contact: Person who was identified as a contact of a primary case during their infectious period (between two days before the date of the case's onset of symptoms or test date, if not symptomatic, and the date of tracing).
Secondary case: Person defined as a primary contact with evidence of infection as determined by detection of SARS-CoV-2 RNA by nucleic acid amplification and had a contact event with the

Supplementary Figure 2: Diagram showing the different definitions for the time taken to initiate and complete contact tracing. The length of time taken for each stage in the diagram is the median time taken for that stage in the control group.

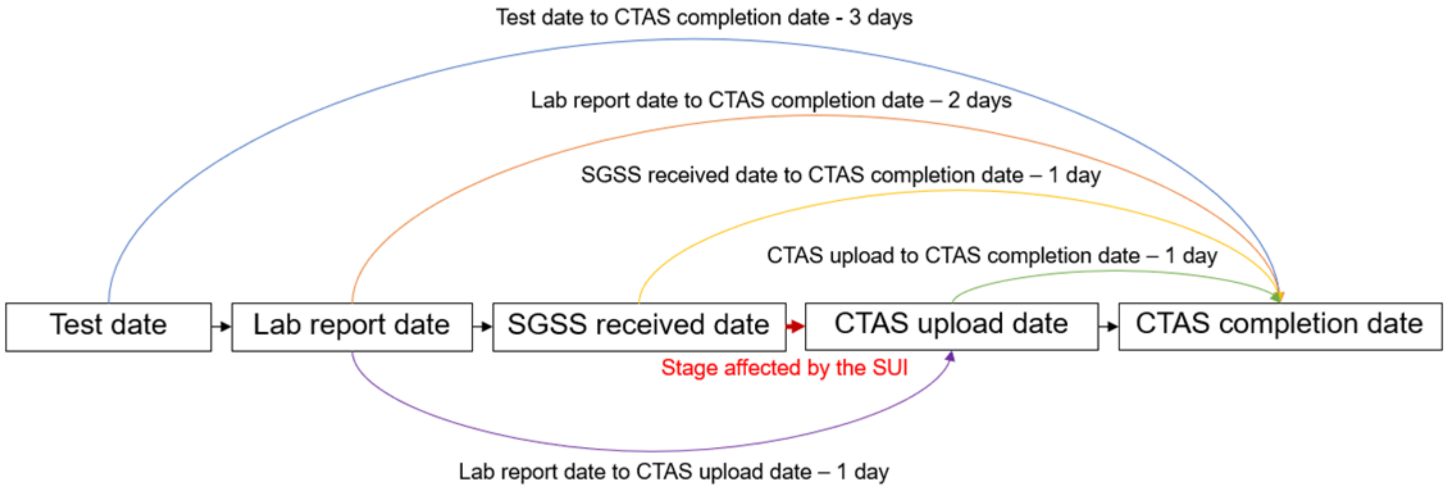

Supplementary Table 1: Description of each step of the process of matching SGSS records to CTAS records

**Field(s) used for matching**	**Records matched (n = 15,861)**
1. CDR Specimen Request SK number*	14,655
2. NHS number, forename, surname, date of birth, postcode	657
3. NHS number, forename, surname, date of birth	64
4. NHS number, date of birth, postcode	18
5. NHS number	1
6. Forename, surname, date of birth, postcode	72
**Total matched**	**15,467 (98%)**

* CDR Specimen Request SK number is the unique number identifier for each SGSS record

Supplementary Figure 3: Data flow chart describing exclusion criteria and de-duplication performed on the dataset of primary cases affected by the contact tracing delay caused by the incident. Only cases arising from community testing (pillar 2 testing) were affected; therefore, non-pillar 2 test episodes (including hospital testing) were excluded from the control group.

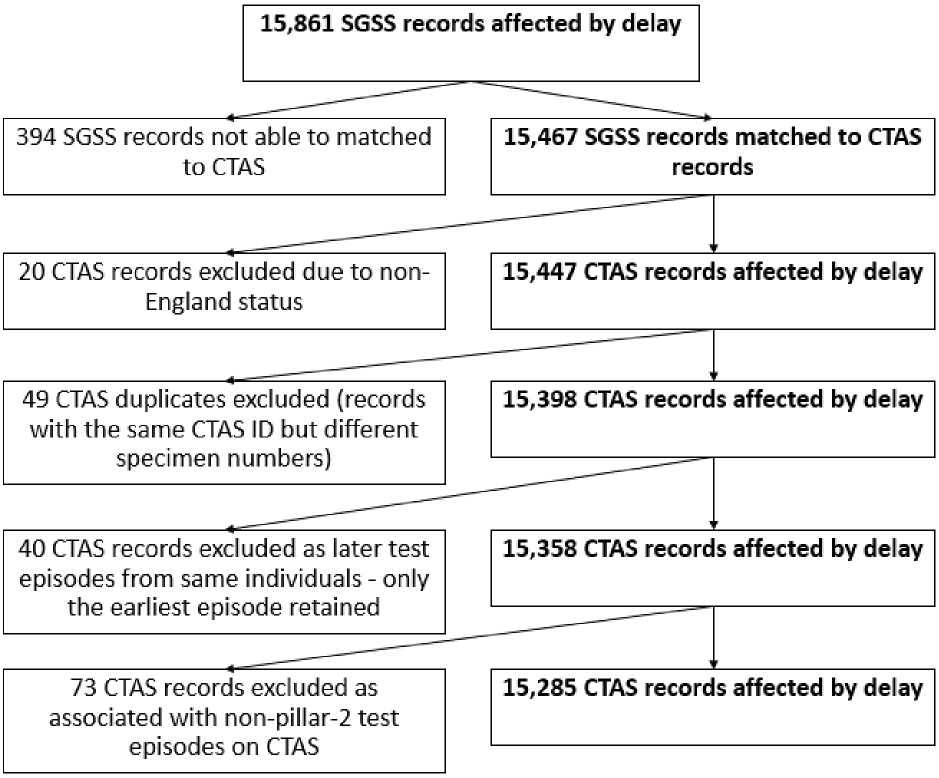
15,861 SGSS records affected by delay
394 SGSS records not able to matched to CTAS
15,467 SGSS records matched to CTAS records
20 CTAS records excluded due to non-England status
15,447 CTAS records affected by delay
49 CTAS duplicates excluded (records with the same CTAS ID but different specimen numbers)
15,398 CTAS records affected by delay
40 CTAS records excluded as later test episodes from same individuals - only the earliest episode retained
15,358 CTAS records affected by delay
73 CTAS records excluded as associated with non-pillar-2 test episodes on CTAS
15,285 CTAS records affected by delay

Supplementary Table 2: Case demographics and contact tracing outcomes

	Delay group			Control group		
**Cases**	**Primary**	**Secondary**	**Tertiary**	**Primary**	**Secondary**	**Tertiary**
Number of observations	15,285	2748	382	43,742	9575	1335
Number of individuals	15,285 (100%)	2695 (98%)	375 (98%)	43,742 (100%)	9382 (98%)	1307 (98%)
Sex						
Male	7300 (48%)	1242 (45%)	165 (43%)	20,674 (47%)	4186 (44%)	606 (45%)
Female	7985 (52%)	1436 (52%)	203 (53%)	23,067 (53%)	5105 (53%)	692 (52%)
NA	0 (0%)	70 (3%)	14 (4%)	1 (0%)	284 (3%)	37 (3%)
Age (years)						
< 18	1360 (9%)	225 (8%)	33 (9%)	3509 (8%)	881 (9%)	158 (12%)
18 – 29	6863 (45%)	1303 (47%)	198 (52%)	19,806 (45%)	4432 (46%)	651 (49%)
30 – 49	4030 (26%)	591 (22%)	75 (20%)	10,951 (25%)	2102 (22%)	257 (19%)
50 – 69	2585 (17%)	556 (20%)	70 (18%)	7800 (18%)	1852 (19%)	234 (18%)
>= 70	447 (3%)	68 (2%)	6 (2%)	1676 (4%)	298 (3%)	33 (3%)
NA	0 (0%)	5 (0%)	0 (0%)	0 (0%)	10 (0%)	2 (0%)
Ethnicity						
Black/ African/ Caribbean/ Black British	258 (2%)	30 (1%)	5 (1%)	804 (2%)	101 (1%)	6 (0%)
Indian (Asian or Asian British)	463 (3%)	90 (3%)	16 (4%)	1420 (3%)	296 (3%)	38 (3%)
Mixed/ Multiple ethnic groups	324 (2%)	51 (2%)	13 (3%)	1071 (2%)	181 (2%)	22 (2%)
Other	156 (1%)	19 (1%)	5 (1%)	402 (1%)	64 (1%)	5 (0%)
Other Asian/ Asian British	435 (3%)	87 (3%)	2 (1%)	1179 (3%)	192 (2%)	24 (2%)
Pakistani (Asian or Asian British)	702 (5%)	91 (3%)	13 (3%)	1829 (4%)	286 (3%)	19 (1%)
White	8525 (56%)	1563 (57%)	196 (51%)	26,100 (60%)	5466 (57%)	748 (56%)
Not stated	4422 (29%)	817 (30%)	132 (35%)	10,937 (25%)	2989 (31%)	473 (35%)
Geographic region						
East Midlands	1042 (7%)	202 (7%)	40 (10%)	3702 (8%)	993 (10%)	170 (13%)
East of England	373 (2%)	74 (3%)	11 (3%)	1234 (3%)	290 (3%)	55 (4%)
London	1174 (8%)	196 (7%)	20 (5%)	3966 (9%)	755 (8%)	111 (8%)
North East	1974 (13%)	338 (12%)	50 (13%)	4209 (10%)	914 (10%)	123 (9%)

For peer review only

North West	5400 (35%)	935 (34%)	110 (29%)	14,679 (34%)	3125 (33%)	372 (28%)
South East	682 (4%)	139 (5%)	23 (6%)	2385 (5%)	489 (5%)	91 (7%)
South West	481 (3%)	95 (3%)	14 (4%)	1718 (4%)	336 (4%)	55 (4%)
West Midlands	1380 (9%)	203 (7%)	27 (7%)	3707 (8%)	791 (8%)	115 (9%)
Yorkshire and the Humber	2713 (18%)	549 (20%)	86 (23%)	7876 (18%)	1837 (19%)	239 (18%)
NA	66 (0%)	17 (1%)	1 (0%)	266 (1%)	45 (1%)	4 (3%)
Completed contact tracing (n (%, 95%CI))						
Yes	12221 (80%, 79-81%)	2137 (78%, 76-79%)	276 (72%, 67-77%)	36,503 (83%, 83-84%)	7291 (76%, 75-77%)	971 (73%, 70-75%)
Difference in proportions who completed contact tracing (delay group minus control group) (95% CI)	-3.5% (-4.2% to -2.8%)	1.6% (-0.2% to 3.4%)	-0.5% (-5.7% to 4.8%)	N/A	N/A	N/A
Median (and IQR) number of contacts reported per case						
All	3 (1-4)	3 (1-4)	3 (1-5)	3 (1-4)	3 (1-4)	3 (1-4)
Household	2 (1-3)	2 (1-3)	3 (1-4)	2 (1-3)	2 (1-4)	2 (1-3)
Non-household	0 (0-1)	0 (0-0)	0 (0-0)	0 (0-1)	0 (0-0)	0 (0-0)
Mean number of contacts reported per case						
All	3.0	3.0	3.6	3.2	3.1	3.2
Household	2.2	2.4	2.6	2.3	2.5	2.4
Non-household	0.8	0.6	1.0	0.9	0.6	0.7
Proportion of cases with ≥2 household contacts (n (%, 95%CI))	7361 (48%, 47-49%)	1348 (49%, 47-51%)	182 (48%, 43-53%)	22,244 (51%, 50-51%)	4717 (49%, 48-50%)	631 (47%, 45-50%)
Median date of test	28/09/2020	01/10/2020	03/10/2020	01/10/20	03/10/2020	05/10/2020
Median (and IQR) time taken to initiate contact tracing, days between (median (IQR)):						

For peer review only

Test date to CTAS completion date	6 (4-7)	4 (2-5)	4 (2-5)	3 (2-5)	3 (2-5)	3 (2-5)
SGSS lab report date to CTAS completion date	4 (3-6)	2 (1-4)	2 (1-4)	1 (1-3)	2 (1-3)	1 (1-3)
SGSS received date to CTAS completion date	2 (1-3)	1 (1-3)	1 (1-3)	1 (1-3)	1 (1-3)	1 (1-2)
CTAS upload date to CTAS completion date	1 (1-2)	1 (0-2)	1 (0-2)	1 (0-2)	1 (0-2)	1 (0-2)
SGSS lab report date to CTAS upload date	3 (2-4)	1 (0-2)	1 (0-1)	1 (0-1)	1 (0-1)	1 (0-1)
Mean time taken for contact tracing, time between:						
SGSS lab report date to CTAS upload date	3.4	1.3	1.1	1.0	0.8	0.8
Test date to CTAS completion date	6.1	4.1	4.2	3.5	3.7	3.7

For peer review only

Supplementary Table 3: Contact demographics and contact tracing outcomes

	Delay group		Control group	
**Contacts**	**Primary**	**Secondary**	**Primary**	**Secondary**
Number of observations	37,373	6540	121,205	23,119
Number of individuals	34,047 (91%)	6045 (92%)	109,907 (91%)	21,201 (92%)
Sex				
Male	8549 (23%)	1486 (23%)	31,318 (26%)	5283 (23%)
Female	10,002 (27%)	1767 (27%)	37,269 (31%)	6260 (27%)
NA	18,822 (50%)	3287 (50%)	52,618 (43%)	11,576 (50%)
Age (years)				
< 18	3000 (8%)	394 (6%)	9989 (8%)	1653 (7%)
18 – 29	7581 (20%)	1548 (24%)	30,151 (24%)	5486 (24%)
30 – 49	4319 (12%)	695 (11%)	15,050 (12%)	2336 (10%)
50 – 69	3480 (9%)	599 (9%)	12,464 (10%)	2011 (9%)
>= 70	428 (1%)	60 (1%)	1875 (2%)	246 (1%)
NA	18,565 (50%)	3244 (50%)	51,706 (43%)	11,387 (49%)
Ethnicity				
Black/ African/ Caribbean/ Black British	360 (1%)	54 (1%)	1352 (1%)	178 (1%)
Indian (Asian or Asian British)	722 (2%)	147 (2%)	2434 (2%)	494 (2%)
Mixed/ Multiple ethnic groups	558 (1%)	90 (1%)	2111 (2%)	331 (1%)
Other	190 (1%)	30 (0%)	636 (1%)	78 (0%)
Other Asian/ Asian British	641 (2%)	124 (2%)	2209 (2%)	329 (1%)
Pakistani (Asian or Asian British)	908 (2%)	139 (2%)	3107 (3%)	428 (2%)
White	14,236 (38%)	2504 (38%)	53,468 (44%)	9122 (39%)
NA	19,758 (53%)	3452 (53%)	55,888 (46%)	12,159 (53%)
Geographic region				
East Midlands	2660 (7%)	543 (8%)	12,198 (10%)	2836 (12%)
East of England	932 (2%)	163 (2%)	3356 (3%)	697 (3%)
London	2469 (7%)	436 (6%)	9614 (8%)	1742 (8%)
North East	4949 (13%)	828 (13%)	11,818 (10%)	2284 (10%)
North West	12,753 (34%)	2150 (33%)	38,641 (32%)	7064 (31%)

For peer review only

South East	1599 (4%)	324 (5%)	6568 (5%)	1087 (5%)
South West	1194 (3%)	229 (4%)	5315 (4%)	781 (3%)
West Midlands	3405 (9%)	486 (7%)	10,175 (8%)	2072 (9%)
Yorkshire and the Humber	7293 (20%)	1348 (21%)	23,002 (19%)	4444 (19%)
NA	119 (0%)	33 (1%)	518 (0%)	112 (0%)
Completed contact tracing (n (%, 95%CI))				
Yes	19,072 (51%, 51-52%)	3361 (51%, 50-53%)	69,702 (58%, 57-58%)	11,915 (52%, 51-52%)
Difference in proportions who completed contact tracing (delay group minus control group) (95% CI)	-6.5% (-7.1% to -5.9%)	-0.1% (-1.5% to 1.2%)	N/A	N/A
Nature of contact (n (%, 95%CI))				
Household	27,477 (74%, 73-74%)	5152 (79%, 78-80%)	85,538 (71%, 70-71%)	18,221 (79%, 78-79%)
Non-household	9,635 (26%, 25-26%)	1,371 (21%, 20-22%)	34,699 (29%, 28-29%)	4609 (20%, 19-20%)
NA	261 (1%)	17 (0%)	968 (1%)	289 (1%)
Median time taken to initiate contact tracing, days between (median (IQR)):				
Test date of associated case and contact's CTAS completion date	6 (5-8)	4 (3-6)	3 (2-5)	4 (3-5)
SGSS lab report date of associated case and contact's completion date	5 (4-7)	3 (1-4)	2 (1-4)	2 (1-4)
SGSS received date of associated case and contact's completion date	3 (1-4)	2 (1-4)	2 (1-3)	2 (1-3)
CTAS upload date of case and contact's completion date	2 (1-3)	2 (1-3)	1 (0-3)	2 (1-3)

For peer review only

CTAS upload date of contact and contact's completion date	1 (0-2)	1 (0-2)	1 (0-1)	1 (0-1)
SGSS lab report date of associated case and contact's CTAS upload date	4 (3-6)	2 (1-4)	1 (1-3)	1 (1-3)
Mean time taken for contact tracing, days between				
SGSS lab report date of associated case and contact's upload date	4.5	2.4	2.0	2.0
Test date of associated case and contact's completion date	6.4	4.4	3.9	4.2
Estimated compliance of cases with self-isolation, for non-household contact events only*				
Contact event date was before test date of associated case	9276 (94%) (n = 9820)	1289 (93%) (n = 1383)	33,284 (94%) (n = 35,368)	4689 (97%) (n = 4852)
Contact event date was before laboratory report date of associated case	9553 (97%) (n = 9816)	1335 (97%) (n = 1377)	34,867 (99%) (n = 35,277)	4744 (98%) (n = 4822)
Contact event date was before symptom onset date of associated case	7245 (78%) (n = 9267)	1069 (83%) (n = 1288)	25,167 (76%) (n = 33,136)	3843 (87%) (n = 4437)

*The compliance of cases with self-isolation was explored by describing whether the date of test, laboratory report, and symptom onset of the case occurred before or after the date of contact with their associated contacts, for non-household contacts only. If the case made non-household contacts after taking a test, developing symptoms, or being informed they had tested positive (using laboratory report date as a proxy), this suggests a lack of compliance with self-isolation.

Supplementary Table 4: Description of the odds ratio for primary and secondary contacts of experiencing health outcomes (mortality or hospitalisation) comparing the delay group to (1) all controls in the control group and (2) to rapidly traced controls in the control group*

	**Comparison of odds in each group***
**Primary contacts**	**Crude odds ratio**
Deaths 28 day definition	
All controls	0.9 (0.4 to 1.6)
Rapidly traced controls	0.7 (0.3 to 1.3)
Deaths 60 day definition	
All controls	0.6 (0.3 to 1.1)
Rapidly traced controls	0.5 (0.2 to 1.0)
Admission to hospital as inpatient	
All controls	1.1 (1.0 to 1.2)
Rapidly traced controls	1.1 (1.0 to 1.1)
**Secondary contacts**	
Deaths 28 day definition	
All controls	0 (NA to Inf)
Rapidly traced controls	0 (NA to Inf)
Deaths 60 day definition	
All controls	0.5 (0.0 to 2.8)
Rapidly traced controls	0.5 (0.0 to 3.4)
Admission to hospital as inpatient	
All controls	1.1 (0.9 to 1.3)
Rapidly traced controls	0.9 (0.8 to 1.1)

*The control group is the reference group (odds ratio = 1), compared to the delay group. The control group consisted of either all controls, or rapidly traced controls, defined as contacts with a date of contact tracing completion within 3 days of the date of test of the associated case.

For peer review only

The RECORD statement – checklist of items, extended from the STROBE statement, that should be reported in observational studies using routinely collected health data.

	Item No.	STROBE items	Location in manuscript where items are reported	RECORD items	Location in manuscript where items are reported
**Title and abstract**					
	1	(a) Indicate the study's design with a commonly used term in the title or the abstract (b) Provide in the abstract an informative and balanced summary of what was done and what was found	1: Page 1, Title and Abstract (Design)	RECORD 1.1: The type of data used should be specified in the title or abstract. When possible, the name of the databases used should be included.<br><br>RECORD 1.2: If applicable, the geographic region and timeframe within which the study took place should be reported in the title or abstract.<br><br>RECORD 1.3: If linkage between databases was conducted for the study, this should be clearly stated in the title or abstract.	1.1: Page 1, Title and Abstract (Objective)<br><br>1.2: Page 1, Title and Abstract (Objective, Design, Setting)<br><br>1.3: Page 1, Abstract (Main outcome measures)
**Introduction**					
Background rationale	2	Explain the scientific background and rationale for the investigation being reported	2: Page 2, Introduction (paragraph 1 and 2)		
Objectives	3	State specific objectives, including any prespecified hypotheses	3: Page 3, Aims and Objectives		
**Methods**					
Study Design	4	Present key elements of study design early in the paper	4: Page 4, Methods (Data sources)		
Setting	5	Describe the setting, locations, and relevant dates, including periods of recruitment, exposure, follow-up, and data collection	5: Page 4-5, Methods (Data sources, Identifying cases and contacts)		

For peer review only

Participants	6	*(a) Cohort study* - Give the eligibility criteria, and the sources and methods of selection of participants. Describe methods of follow-up<br>*Case-control study* - Give the eligibility criteria, and the sources and methods of case ascertainment and control selection. Give the rationale for the choice of cases and controls<br>*Cross-sectional study* - Give the eligibility criteria, and the sources and methods of selection of participants<br><br>*(b) Cohort study* - For matched studies, give matching criteria and number of exposed and unexposed<br>*Case-control study* - For matched studies, give matching criteria and the number of controls per case	6: Page 4-5, Methods (Data sources, Identifying cases and contacts)	RECORD 6.1: The methods of study population selection (such as codes or algorithms used to identify subjects) should be listed in detail. If this is not possible, an explanation should be provided.<br><br>RECORD 6.2: Any validation studies of the codes or algorithms used to select the population should be referenced. If validation was conducted for this study and not published elsewhere, detailed methods and results should be provided.<br><br>RECORD 6.3: If the study involved linkage of databases, consider use of a flow diagram or other graphical display to demonstrate the data linkage process, including the number of individuals with linked data at each stage.	6.1: Page 4-5, Methods (Data sources, Identifying cases and contacts) and Supplementary Material, Page 1-2 (Supplementary Figure 1, Supplementary Table 1)<br><br>6.2: Page 4-5, Methods (Data sources, Identifying cases and contacts) and Supplementary Material, Page 3 (Supplementary Figure 3)<br><br>6.3: Page 4, Methods (Data sources, Identifying cases and contacts, Linkage to health outcomes) and Supplementary Material, Page 2-3 (Supplementary Table 1, Supplementary Figure 3)
Variables	7	Clearly define all outcomes, exposures, predictors, potential confounders, and effect	7: Page 5, Methods (Regression analysis)	RECORD 7.1: A complete list of codes and algorithms used to classify exposures, outcomes, confounders, and effect modifiers should be provided. If	7.1: Page 5, Methods (Regression analysis)

For peer review only

		modifiers. Give diagnostic criteria, if applicable.		these cannot be reported, an explanation should be provided.	
Data sources/ measurement	8	For each variable of interest, give sources of data and details of methods of assessment (measurement).<br>Describe comparability of assessment methods if there is more than one group	8: Page 5, Methods (Descriptive analysis)		
Bias	9	Describe any efforts to address potential sources of bias	9: Pages 4-5, Methods (Data sources, Regression analysis)		
Study size	10	Explain how the study size was arrived at	10: Page 4, Methods (Data sources, Identifying cases and contacts)		
Quantitative variables	11	Explain how quantitative variables were handled in the analyses. If applicable, describe which groupings were chosen, and why	11: Page 5, Methods (Descriptive analysis, Regression analysis)		
Statistical methods	12	(a) Describe all statistical methods, including those used to control for confounding<br>(b) Describe any methods used to examine subgroups and interactions<br>(c) Explain how missing data were addressed<br>(d) *Cohort study* - If applicable, explain how loss to follow-up was addressed<br>*Case-control study* - If applicable, explain how matching of cases and controls was addressed	12: Page 4-5, Methods (Descriptive analysis, Regression analysis)		

For peer review only

		Cross-sectional study - If applicable, describe analytical methods taking account of sampling strategy<br>(e) Describe any sensitivity analyses			
Data access and cleaning methods		..		RECORD 12.1: Authors should describe the extent to which the investigators had access to the database population used to create the study population.<br><br>RECORD 12.2: Authors should provide information on the data cleaning methods used in the study.	12.1: Page 5, Methods (Descriptive analysis)<br><br>12.2: Supplementary Material, Page 2-3 (Supplementary Figure 3)
Linkage		..		RECORD 12.3: State whether the study included person-level, institutional-level, or other data linkage across two or more databases. The methods of linkage and methods of linkage quality evaluation should be provided.	12.3: Page 4, Methods (Data sources, Linkage to health outcomes)
**Results**					
Participants	13	(a) Report the numbers of individuals at each stage of the study (*e.g.*, numbers potentially eligible, examined for eligibility, confirmed eligible, included in the study, completing follow-up, and analysed)<br>(b) Give reasons for non-participation at each stage.<br>(c) Consider use of a flow diagram	13: Page 6, Results (Sample characteristics, Table 1, Table 2) and Supplementary Material, Pages 3-9 (Supp. Figure 3, Supp. Tables 2 and 3)	RECORD 13.1: Describe in detail the selection of the persons included in the study (*i.e.,* study population selection) including filtering based on data quality, data availability and linkage. The selection of included persons can be described in the text and/or by means of the study flow diagram.	13.1: Page 6, Results (Sample characteristics, Table 1, Table 2) and Supplementary Material, Pages 3-9 (Supp. Figure 3, Supp. Tables 2 and 3)
Descriptive data	14	(a) Give characteristics of study participants (*e.g.*, demographic, clinical, social) and information	14: Pages 7-11, Results (Tables 1, 2 and 4) and		

For peer review - only

		on exposures and potential confounders<br>(b) Indicate the number of participants with missing data for each variable of interest<br>(c) *Cohort study* - summarise follow-up time (*e.g.*, average and total amount)	Supplementary Material, Pages 4-9 (Supp. Tables 2 and 3)		
Outcome data	15	*Cohort study* - Report numbers of outcome events or summary measures over time<br>*Case-control study* - Report numbers in each exposure category, or summary measures of exposure<br>*Cross-sectional study* - Report numbers of outcome events or summary measures	15: Pages 7-11, Results (Tables 1, 2, 3 and 4)		
Main results	16	(a) Give unadjusted estimates and, if applicable, confounder-adjusted estimates and their precision (e.g., 95% confidence interval). Make clear which confounders were adjusted for and why they were included<br>(b) Report category boundaries when continuous variables were categorized<br>(c) If relevant, consider translating estimates of relative risk into absolute risk for a meaningful time period	16: Page 11, Results (Tables 3 and 4)		
Other analyses	17	Report other analyses done—e.g., analyses of subgroups and interactions, and sensitivity analyses	17: Supplementary Material, Page 10 (Supp. Table 4)		
**Discussion**					

For peer review only

Key results	18	Summarise key results with reference to study objectives	18: Page 13, Discussion (Statement of principal findings)		
Limitations	19	Discuss limitations of the study, taking into account sources of potential bias or imprecision. Discuss both direction and magnitude of any potential bias	19: Pages 13-14, Discussion (Strengths and weaknesses, Strengths and weaknesses in relation to other studies)	RECORD 19.1: Discuss the implications of using data that were not created or collected to answer the specific research question(s). Include discussion of misclassification bias, unmeasured confounding, missing data, and changing eligibility over time, as they pertain to the study being reported.	19: Pages 13-14, Discussion (Strengths and weaknesses, Strengths and weaknesses in relation to other studies)
Interpretation	20	Give a cautious overall interpretation of results considering objectives, limitations, multiplicity of analyses, results from similar studies, and other relevant evidence	20: Pages 14-15, Discussion (Strengths and weaknesses in relation to other studies, Meaning of the study, Conclusions)		
Generalisability	21	Discuss the generalisability (external validity) of the study results	21: Page 14-15, Discussion (Meaning of the study, Unanswered questions and future research)		
**Other Information**					
Funding	22	Give the source of funding and the role of the funders for the present study and, if applicable, for the original study on which the present article is based	22: Page 15, Funding		
Accessibility of protocol, raw data, and programming code		..		RECORD 22.1: Authors should provide information on how to access any supplemental information such as the study protocol, raw data, or programming code.	22.1: Page 16, Data availability statement

For peer review only

*Reference: Benchimol EI, Smeeth L, Guttmann A, Harron K, Moher D, Petersen I, Sørensen HT, von Elm E, Langan SM, the RECORD Working Committee. The REporting of studies Conducted using Observational Routinely-collected health Data (RECORD) Statement. *PLoS Medicine* 2015; in press.

*Checklist is protected under Creative Commons Attribution (CC BY) license.

For peer review only
