# Supplementary material for: Evaluating the impact on health outcomes of an event that resulted in a delay in contact tracing of COVID-19 cases in England, September 2020: an observational study
Source: BMJ Open. 2023 Oct 12;13(10):e064982. doi: 10.1136/bmjopen-2022-064982 (PMC10583033; doi:10.1136/bmjopen-2022-064982)
Supplement: Supplementary file 3 [file bmjopen-2022-064982supp001.pdf]

Supplementary Material

Supplementary Figure 1: Definitions of SARS-CoV-2 cases and contacts

Definitions of cases and contacts in the Contact Tracing Advisory Service (CTAS) database
Individual records in the data represent case or contact episodes, where a case corresponds to a person with a positive test for SARS-CoV-2 and no case report in the 14 days prior. A contact is a person who was named by a case as being a contact between two days before the date of the case's onset of symptoms (or test date, if not symptomatic) and the date of tracing. This includes being a member of or visitor to the case's household, or close contact during an activity or in a work or education setting. An episode is an individual entry in NHS Test and Trace based on one of the above two definitions.

Delay group
Primary case: Confirmed case with laboratory evidence of infection as determined by detection of SARS-CoV-2 RNA by nucleic acid amplification, who had no case report in the 14 days prior to their test date, and who was known from SGSS records to have been impacted by the serious untoward incident (which affected some case records from 25 September to 2 October 2020).
Primary contact: Person who was identified as a contact of a primary case during their infectious period (between two days before the date of the case's onset of symptoms or test date, if not symptomatic, and the date of tracing).
Secondary case: Person defined as a primary contact with evidence of infection as determined by detection of SARS-CoV-2 RNA by nucleic acid amplification and had a contact event with the primary case between two days and 14 days inclusive prior to the onset of symptoms (or test date if no symptom onset available) of the secondary case.
Secondary contact: Person who was identified as a contact of a secondary case during their infectious period (between two days before the date of the case's onset of symptoms or test date, if not symptomatic, and the date of tracing).
Tertiary case: Person defined as a secondary contact with evidence of infection as determined by detection of SARS-CoV-2 RNA by nucleic acid amplification and had a contact event with the primary case between two days and 14 days inclusive prior to the onset of symptoms (or test date if no symptom onset available) of the tertiary case.

Control group
Primary case: Confirmed case with laboratory evidence of infection as determined by detection of SARS-CoV-2 RNA by nucleic acid amplification, who had no case report in the 14 days prior to their test date, and who was not on the list of case records affected by the incident, but had a CTAS upload date from within the time period of the incident (25 September to 2 October 2020 inclusive).
Primary contact: Person who was identified as a contact of a primary case during their infectious period (between two days before the date of the case's onset of symptoms or test date, if not symptomatic, and the date of tracing).
Secondary case: Person defined as a primary contact with evidence of infection as determined by detection of SARS-CoV-2 RNA by nucleic acid amplification and had a contact event with the

Supplementary Figure 2: Diagram showing the different definitions for the time taken to initiate and complete contact tracing. The length of time taken for each stage in the diagram is the median time taken for that stage in the control group.

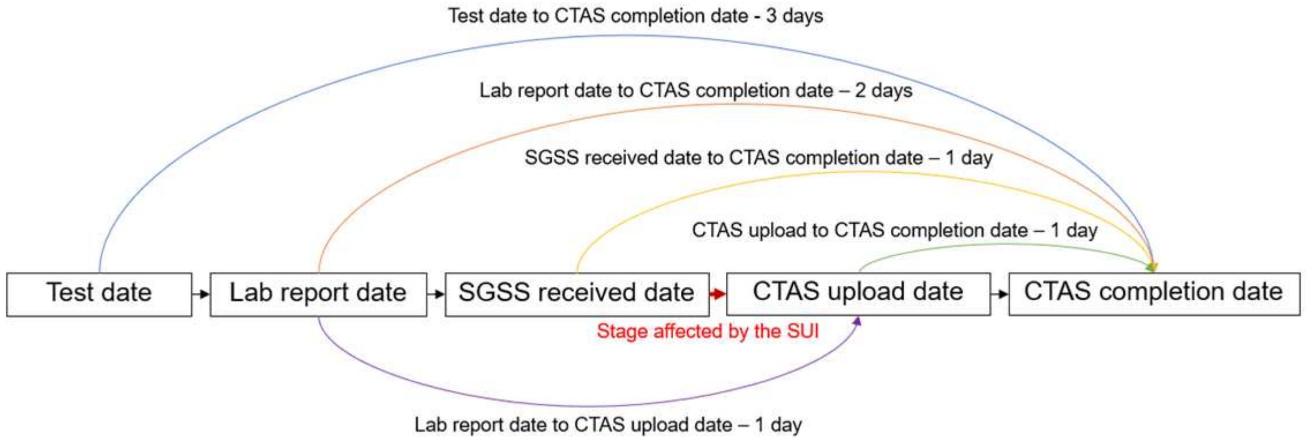

Supplementary Table 1: Description of each step of the process of matching SGSS records to CTAS records

**Field(s) used for matching**	**Records matched (n = 15,861)**
1. CDR Specimen Request SK number*	14,655
2. NHS number, forename, surname, date of birth, postcode	657
3. NHS number, forename, surname, date of birth	64
4. NHS number, date of birth, postcode	18
5. NHS number	1
6. Forename, surname, date of birth, postcode	72
**Total matched**	**15,467 (98%)**

* CDR Specimen Request SK number is the unique number identifier for each SGSS record

Supplementary Figure 3: Data flow chart describing exclusion criteria and de-duplication performed on the dataset of primary cases affected by the contact tracing delay caused by the incident. Only cases arising from community testing (pillar 2 testing) were affected; therefore, non-pillar 2 test episodes (including hospital testing) were excluded from the control group.

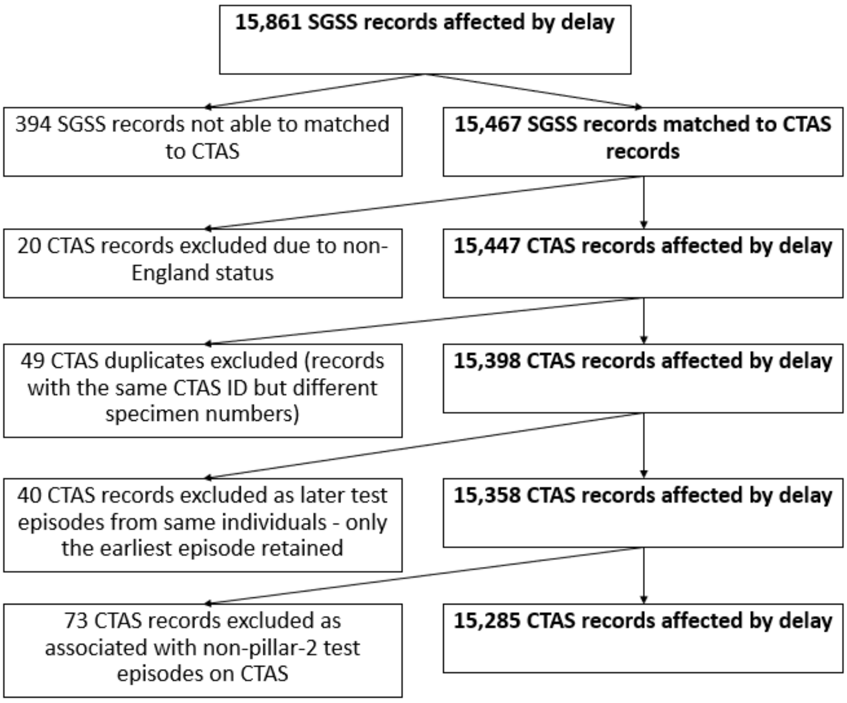
15,861 SGSS records affected by delay
394 SGSS records not able to matched to CTAS
15,467 SGSS records matched to CTAS records
20 CTAS records excluded due to non-England status
15,447 CTAS records affected by delay
49 CTAS duplicates excluded (records with the same CTAS ID but different specimen numbers)
15,398 CTAS records affected by delay
40 CTAS records excluded as later test episodes from same individuals - only the earliest episode retained
15,358 CTAS records affected by delay
73 CTAS records excluded as associated with non-pillar-2 test episodes on CTAS
15,285 CTAS records affected by delay

Supplementary Table 2: Case demographics and contact tracing outcomes

	**Delay group**			**Control group**		
**Cases**	**Primary**	**Secondary**	**Tertiary**	**Primary**	**Secondary**	**Tertiary**
Number of observations	15,285	2748	382	43,742	9575	1335
Number of individuals	15,285 (100%)	2695 (98%)	375 (98%)	43,742 (100%)	9382 (98%)	1307 (98%)
Sex						
Male	7300 (48%)	1242 (45%)	165 (43%)	20,674 (47%)	4186 (44%)	606 (45%)
Female	7985 (52%)	1436 (52%)	203 (53%)	23,067 (53%)	5105 (53%)	692 (52%)
NA	0 (0%)	70 (3%)	14 (4%)	1 (0%)	284 (3%)	37 (3%)
Age (years)						
< 18	1360 (9%)	225 (8%)	33 (9%)	3509 (8%)	881 (9%)	158 (12%)
18 – 29	6863 (45%)	1303 (47%)	198 (52%)	19,806 (45%)	4432 (46%)	651 (49%)
30 – 49	4030 (26%)	591 (22%)	75 (20%)	10,951 (25%)	2102 (22%)	257 (19%)
50 – 69	2585 (17%)	556 (20%)	70 (18%)	7800 (18%)	1852 (19%)	234 (18%)
>= 70	447 (3%)	68 (2%)	6 (2%)	1676 (4%)	298 (3%)	33 (3%)
NA	0 (0%)	5 (0%)	0 (0%)	0 (0%)	10 (0%)	2 (0%)
Ethnicity						
Black/ African/ Caribbean/ Black British	258 (2%)	30 (1%)	5 (1%)	804 (2%)	101 (1%)	6 (0%)
Indian (Asian or Asian British)	463 (3%)	90 (3%)	16 (4%)	1420 (3%)	296 (3%)	38 (3%)
Mixed/ Multiple ethnic groups	324 (2%)	51 (2%)	13 (3%)	1071 (2%)	181 (2%)	22 (2%)
Other	156 (1%)	19 (1%)	5 (1%)	402 (1%)	64 (1%)	5 (0%)
Other Asian/ Asian British	435 (3%)	87 (3%)	2 (1%)	1179 (3%)	192 (2%)	24 (2%)
Pakistani (Asian or Asian British)	702 (5%)	91 (3%)	13 (3%)	1829 (4%)	286 (3%)	19 (1%)
White	8525 (56%)	1563 (57%)	196 (51%)	26,100 (60%)	5466 (57%)	748 (56%)
Not stated	4422 (29%)	817 (30%)	132 (35%)	10,937 (25%)	2989 (31%)	473 (35%)
Geographic region						
East Midlands	1042 (7%)	202 (7%)	40 (10%)	3702 (8%)	993 (10%)	170 (13%)
East of England	373 (2%)	74 (3%)	11 (3%)	1234 (3%)	290 (3%)	55 (4%)
London	1174 (8%)	196 (7%)	20 (5%)	3966 (9%)	755 (8%)	111 (8%)
North East	1974 (13%)	338 (12%)	50 (13%)	4209 (10%)	914 (10%)	123 (9%)

North West	5400 (35%)	935 (34%)	110 (29%)	14,679 (34%)	3125 (33%)	372 (28%)
South East	682 (4%)	139 (5%)	23 (6%)	2385 (5%)	489 (5%)	91 (7%)
South West	481 (3%)	95 (3%)	14 (4%)	1718 (4%)	336 (4%)	55 (4%)
West Midlands	1380 (9%)	203 (7%)	27 (7%)	3707 (8%)	791 (8%)	115 (9%)
Yorkshire and the Humber	2713 (18%)	549 (20%)	86 (23%)	7876 (18%)	1837 (19%)	239 (18%)
NA	66 (0%)	17 (1%)	1 (0%)	266 (1%)	45 (1%)	4 (3%)
Completed contact tracing (n (%, 95%CI))						
Yes	12221 (80%, 79-81%)	2137 (78%, 76-79%)	276 (72%, 67-77%)	36,503 (83%, 83-84%)	7291 (76%, 75-77%)	971 (73%, 70-75%)
Difference in proportions who completed contact tracing (delay group minus control group) (95% CI)	-3.5% (-4.2% to -2.8%)	1.6% (-0.2% to 3.4%)	-0.5% (-5.7% to 4.8%)	N/A	N/A	N/A
Median (and IQR) number of contacts reported per case						
All	3 (1-4)	3 (1-4)	3 (1-5)	3 (1-4)	3 (1-4)	3 (1-4)
Household	2 (1-3)	2 (1-3)	3 (1-4)	2 (1-3)	2 (1-4)	2 (1-3)
Non-household	0 (0-1)	0 (0-0)	0 (0-0)	0 (0-1)	0 (0-0)	0 (0-0)
Mean number of contacts reported per case						
All	3.0	3.0	3.6	3.2	3.1	3.2
Household	2.2	2.4	2.6	2.3	2.5	2.4
Non-household	0.8	0.6	1.0	0.9	0.6	0.7
Proportion of cases with ≥2 household contacts (n (%, 95%CI))	7361 (48%, 47-49%)	1348 (49%, 47-51%)	182 (48%, 43-53%)	22,244 (51%, 50-51%)	4717 (49%, 48-50%)	631 (47%, 45-50%)
Median date of test	28/09/2020	01/10/2020	03/10/2020	01/10/20	03/10/2020	05/10/2020
Median (and IQR) time taken to initiate contact tracing, days between (median (IQR)):						

Test date to CTAS completion date	6 (4-7)	4 (2-5)	4 (2-5)	3 (2-5)	3 (2-5)	3 (2-5)
SGSS lab report date to CTAS completion date	4 (3-6)	2 (1-4)	2 (1-4)	1 (1-3)	2 (1-3)	1 (1-3)
SGSS received date to CTAS completion date	2 (1-3)	1 (1-3)	1 (1-3)	1 (1-3)	1 (1-3)	1 (1-2)
CTAS upload date to CTAS completion date	1 (1-2)	1 (0-2)	1 (0-2)	1 (0-2)	1 (0-2)	1 (0-2)
SGSS lab report date to CTAS upload date	3 (2-4)	1 (0-2)	1 (0-1)	1 (0-1)	1 (0-1)	1 (0-1)
Mean time taken for contact tracing, time between:						
SGSS lab report date to CTAS upload date	3.4	1.3	1.1	1.0	0.8	0.8
Test date to CTAS completion date	6.1	4.1	4.2	3.5	3.7	3.7

Supplementary Table 3: Contact demographics and contact tracing outcomes

	**Delay group**		**Control group**	
**Contacts**	**Primary**	**Secondary**	**Primary**	**Secondary**
Number of observations	37,373	6540	121,205	23,119
Number of individuals	34,047 (91%)	6045 (92%)	109,907 (91%)	21,201 (92%)
Sex				
Male	8549 (23%)	1486 (23%)	31,318 (26%)	5283 (23%)
Female	10,002 (27%)	1767 (27%)	37,269 (31%)	6260 (27%)
NA	18,822 (50%)	3287 (50%)	52,618 (43%)	11,576 (50%)
Age (years)				
< 18	3000 (8%)	394 (6%)	9989 (8%)	1653 (7%)
18 – 29	7581 (20%)	1548 (24%)	30,151 (24%)	5486 (24%)
30 – 49	4319 (12%)	695 (11%)	15,050 (12%)	2336 (10%)
50 – 69	3480 (9%)	599 (9%)	12,464 (10%)	2011 (9%)
>= 70	428 (1%)	60 (1%)	1875 (2%)	246 (1%)
NA	18,565 (50%)	3244 (50%)	51,706 (43%)	11,387 (49%)
Ethnicity				
Black/ African/ Caribbean/ Black British	360 (1%)	54 (1%)	1352 (1%)	178 (1%)
Indian (Asian or Asian British)	722 (2%)	147 (2%)	2434 (2%)	494 (2%)
Mixed/ Multiple ethnic groups	558 (1%)	90 (1%)	2111 (2%)	331 (1%)
Other	190 (1%)	30 (0%)	636 (1%)	78 (0%)
Other Asian/ Asian British	641 (2%)	124 (2%)	2209 (2%)	329 (1%)
Pakistani (Asian or Asian British)	908 (2%)	139 (2%)	3107 (3%)	428 (2%)
White	14,236 (38%)	2504 (38%)	53,468 (44%)	9122 (39%)
NA	19,758 (53%)	3452 (53%)	55,888 (46%)	12,159 (53%)
Geographic region				
East Midlands	2660 (7%)	543 (8%)	12,198 (10%)	2836 (12%)
East of England	932 (2%)	163 (2%)	3356 (3%)	697 (3%)
London	2469 (7%)	436 (6%)	9614 (8%)	1742 (8%)
North East	4949 (13%)	828 (13%)	11,818 (10%)	2284 (10%)
North West	12,753 (34%)	2150 (33%)	38,641 (32%)	7064 (31%)

South East	1599 (4%)	324 (5%)	6568 (5%)	1087 (5%)
South West	1194 (3%)	229 (4%)	5315 (4%)	781 (3%)
West Midlands	3405 (9%)	486 (7%)	10,175 (8%)	2072 (9%)
Yorkshire and the Humber	7293 (20%)	1348 (21%)	23,002 (19%)	4444 (19%)
NA	119 (0%)	33 (1%)	518 (0%)	112 (0%)
Completed contact tracing (n (%, 95%CI))				
Yes	19,072 (51%, 51-52%)	3361 (51%, 50-53%)	69,702 (58%, 57-58%)	11,915 (52%, 51-52%)
Difference in proportions who completed contact tracing (delay group minus control group) (95% CI)	-6.5% (-7.1% to -5.9%)	-0.1% (-1.5% to 1.2%)	N/A	N/A
Nature of contact (n (%, 95%CI))				
Household	27,477 (74%, 73-74%)	5152 (79%, 78-80%)	85,538 (71%, 70-71%)	18,221 (79%, 78-79%)
Non-household	9,635 (26%, 25-26%)	1,371 (21%, 20-22%)	34,699 (29%, 28-29%)	4609 (20%, 19-20%)
NA	261 (1%)	17 (0%)	968 (1%)	289 (1%)
Median time taken to initiate contact tracing, days between (median (IQR)):				
Test date of associated case and contact's CTAS completion date	6 (5-8)	4 (3-6)	3 (2-5)	4 (3-5)
SGSS lab report date of associated case and contact's completion date	5 (4-7)	3 (1-4)	2 (1-4)	2 (1-4)
SGSS received date of associated case and contact's completion date	3 (1-4)	2 (1-4)	2 (1-3)	2 (1-3)
CTAS upload date of case and contact's completion date	2 (1-3)	2 (1-3)	1 (0-3)	2 (1-3)

CTAS upload date of contact and contact's completion date	1 (0-2)	1 (0-2)	1 (0-1)	1 (0-1)
SGSS lab report date of associated case and contact's CTAS upload date	4 (3-6)	2 (1-4)	1 (1-3)	1 (1-3)
Mean time taken for contact tracing, days between				
SGSS lab report date of associated case and contact's upload date	4.5	2.4	2.0	2.0
Test date of associated case and contact's completion date	6.4	4.4	3.9	4.2
Estimated compliance of cases with self-isolation, for non-household contact events only*				
Contact event date was before test date of associated case	9276 (94%) (n = 9820)	1289 (93%) (n = 1383)	33,284 (94%) (n = 35,368)	4689 (97%) (n = 4852)
Contact event date was before laboratory report date of associated case	9553 (97%) (n = 9816)	1335 (97%) (n = 1377)	34,867 (99%) (n = 35,277)	4744 (98%) (n = 4822)
Contact event date was before symptom onset date of associated case	7245 (78%) (n = 9267)	1069 (83%) (n = 1288)	25,167 (76%) (n = 33,136)	3843 (87%) (n = 4437)

*The compliance of cases with self-isolation was explored by describing whether the date of test, laboratory report, and symptom onset of the case occurred before or after the date of contact with their associated contacts, for non-household contacts only. If the case made non-household contacts after taking a test, developing symptoms, or being informed they had tested positive (using laboratory report date as a proxy), this suggests a lack of compliance with self-isolation.

Supplementary Table 4: Description of the odds ratio for primary and secondary contacts of experiencing health outcomes (mortality or hospitalisation) comparing the delay group to (1) all controls in the control group and (2) to rapidly traced controls in the control group*

	**Comparison of odds in each group***
**Primary contacts**	**Crude odds ratio**
Deaths 28 day definition	
All controls	0.9 (0.4 to 1.6)
Rapidly traced controls	0.7 (0.3 to 1.3)
Deaths 60 day definition	
All controls	0.6 (0.3 to 1.1)
Rapidly traced controls	0.5 (0.2 to 1.0)
Admission to hospital as inpatient	
All controls	1.1 (1.0 to 1.2)
Rapidly traced controls	1.1 (1.0 to 1.1)
**Secondary contacts**	
Deaths 28 day definition	
All controls	0 (NA to Inf)
Rapidly traced controls	0 (NA to Inf)
Deaths 60 day definition	
All controls	0.5 (0.0 to 2.8)
Rapidly traced controls	0.5 (0.0 to 3.4)
Admission to hospital as inpatient	
All controls	1.1 (0.9 to 1.3)
Rapidly traced controls	0.9 (0.8 to 1.1)

*The control group is the reference group (odds ratio = 1), compared to the delay group. The control group consisted of either all controls, or rapidly traced controls, defined as contacts with a date of contact tracing completion within 3 days of the date of test of the associated case.
